# The m6A modification-mediated positive feedback between glycolytic lncRNA SLC2A1-DT and c-Myc promotes tumorigenesis of hepatocellular carcinoma

**DOI:** 10.7150/ijbs.86658

**Published:** 2024-02-25

**Authors:** Zhu Zeng, Jie Wang, Fengyu Xu, Ping Hu, Yuhang Hu, Wenfeng Zhuo, Ding Chen, Shengbo Han, Fan Wang, Yong Zhao, Yan Huang, Gang Zhao

**Affiliations:** 1 Department of Emergency Surgery, Union Hospital, Tongji Medical College, Huazhong University of Science and Technology, Wuhan 430022, China.; 2 Department of Emergency Medicine, Union Hospital, Tongji Medical College, Huazhong University of Science and Technology, Wuhan 430022, China.

**Keywords:** Hepatocellular carcinoma, m6A modification, SLC2A1-DT, β-catenin, c-Myc, Glycolysis.

## Abstract

Glycolysis exerts a key role in the metabolic reprogramming of cancer. Specific long non-coding RNAs (lncRNAs) have been identified to exhibit oncogenic glycolysis regulation. Nevertheless, the precise mechanisms by which glycolysis-related lncRNAs control hepatocellular carcinoma (HCC) are still unknown. We profiled and analyzed glycolysis-associated lncRNA signatures using HCC specimens from The Cancer Genome Atlas (TCGA) dataset. Considerable upregulation of the glycolysis-related lncRNA SLC2A1-DT was noted in HCC tissues; this upregulation was strongly linked with advanced tumor stage and poor prognosis. Cell culture and animal-related studies indicated that knockdown or overexpression of SLC2A1-DT obviously restrained or promoted glycolysis, propagation, and metastasis in HCC cells. Mechanistically, SLC2A1-DT enhanced the interaction of protein between β-catenin and YWHAZ, suppressing the binding between β-catenin and β-TrCP, an E3 ubiquitin ligase. Thereby, SLC2A1-DT impeded the β-TrCP-dependent ubiquitination and β-catenin degradation. The upregulated β-catenin activated the transcription of c-Myc, which then increased the transcription of glycolytic genes including SLC2A1, LDHA, and HK2. Additionally, we revealed that c-Myc transcriptionally induced the expression of methyltransferase 3 (METTL3), which increased N6-methyladenosine (m6A) modification and stability of SLC2A1-DT in a YTHDF1 dependent manner. Collectively, we show that the lncRNA SLC2A1-DT promotes glycolysis and HCC tumorigenesis by a m6A modification-mediated positive feedback mechanism with glycolytic regulator c-Myc and suggested as an innovative treatment option and indicator for HCC.

## Introduction

Hepatocellular carcinoma (HCC) represents a serious health concern globally 1. In 2018, about 782,000 cases died due to HCC and 841,000 new cases were reported 2. Despite the abundance of various therapeutic alternatives such as liver transplantation, locoregional therapy and systemic therapy, and surgical harvesting, the rate of 5-year patient' expectancy post- surgery persists to be ineffective 3. Thus, it is urgent to recognize therapeutic targets and effective biomarkers for HCC patients. To meet the needs of tumor growth, cancer cells primarily utilize the aerobic glycolytic pathway to metabolize glucose and generate ATP, referred to as the “Warburg effect” 4. HCC is known to have a high rate of glucose metabolism, which is well supported by the correlation between high accumulation of 18F-fludeoxyglucose (18F-FDG) and unfavorable histopathologic features, validated by positron emission tomography (PET) 5, 6. Enhanced glycolysis is a hallmark of HCC and is associated with proliferation, invasion, and metastasis 7. Therefore, further investigation of the mechanism regulating glycolysis in HCC may provide novel therapeutic targets.

Previous studies have identified lncRNAs that exceed 200 nucleotides in length as key regulators during tumor progression, including cell proliferation, invasion, metastasis, immune response, and apoptosis 8, 9. Accumulating studies have reported that the link between lncRNAs and aerobic glycolysis may identify novel lncRNAs as therapeutic targets for various cancers 10. For example, the lncRNA-p21 functions as a hypoxia-dependent lncRNA that disrupts the binding of HIF-1α to VHL, subsequently stabilizing HIF-1α and promoting glycolysis in cancer cells 11. Depletion of the lncRNA NBR2 attenuated SLC2A1 expression, thereby reducing cellular glucose uptake 12. Besides, the lncRNA GCASPC destabilizes pyruvate carboxylase, thereby reducing its expression level and activity and suppressing the proliferation of gallbladder cancer cells 13. Nevertheless, the mechanism associated with the regulation of glycolysis by lncRNAs in HCC has not been investigated in depth.

As the most common RNA modification, N6-methyladenosine (m6A) methylation of RNA has been reported as a novel posttranscriptional modification 14, 15. Methylases and demethylases mediate m6A modification of RNA. Besides, several binding proteins regulate the biological functions and fate of m6A-modified RNAs and contribute to the control of RNA splicing, stability, localization, translation, and their interaction with other proteins 16, 17. A growing number of studies have demonstrated that m6A modification, which is the most common intrinsic epigenetic modification of eukaryotic mRNA, has a crucial role in lncRNA life cycle regulation 18. Recently, a study demonstrated that m6A modification upregulated LINC00958 by increasing its stability 19. Another study has shown that m6A is highly enriched on RP11 and increases its nuclear accumulation 20. Consequently, it is important to determine whether m6A modification is implicated in the controlling of lncRNA, glycolysis, or HCC development.

Here, we integrated the HCC datasets from TCGA to indicate that SLC2A1-DT, a glycolysis-related lncRNA, was overexpressed and linked with adverse outcomes in HCC patients. Subsequently, we identified that SLC2A1-DT promoted glycolysis, proliferative, and invasive ability in HCC cells by stabilizing β-catenin protein, which transcriptionally induced the expression of glycolytic genes. Additionally, a sequence of molecular biology examinations was undertaken to elucidate the mechanism by which β-catenin is stabilized via SLC2A1-DT. Meanwhile, we found that c-Myc, the target of β-catenin, reciprocally stabilized SLC2A1-DT. Since online informative analysis indicated a potential m6A modification on the transcript of SLC2A1-DT, we performed methylated RNA immunoprecipitation (MeRIP) assay to explore whether c-Myc stabilized SLC2A1-DT via METTL3-mediated m6A modification. Furthermore, an animal model was developed using nude mice to observe further the contribution of the SLC2A1-DT/β-catenin axis on glycolysis and tumorigenesis of HCC.

## Materials and Methods

### Patient specimens

All clinical specimens were obtained from the Department of Hepatobiliary Surgery, Wuhan Union Medical College Hospital. Before obtaining the clinical specimens, the physician disclosed all the necessary information to the patient and their family and obtained the informed consent form. We selected 32 patients with HCC by random sampling and collected their cancer and corresponding paracancerous tissues. In addition, none of the enrolled patients received radiotherapy or chemotherapy. In the process of specimen collection, we strictly followed the relevant rules and regulations of the hospital and laboratory and obtained the application consent for specimen collection in the operating room. After obtaining the tissue specimens with the help of the operating room physician, we immediately placed them in the liquid nitrogen tank for freezing and long-term registered storage and took them out for subsequent analysis. In this study, the Declaration of Helsinki was followed, and approval was received from the Medical Research Ethics Committee of Huazhong University of Science and Technology (Wuhan, China).

### Cell culture and propagation

HepG2/Hep3B cells were acquired from Genechem Co. (Shanghai, China). MHCC-97H cells were obtained from Wuhan Union Hospital Cancer Center (UHCC, China). BeNa Culture Collection (BNCC, China) provided SMMC-7721/Huh-7 cells. LO2 was obtained from Dr. Q.Y. Ge (Tongji Hospital, Wuhan, China). Cells were grown in DMEM enriched with 10% fetal bovine serum (Gibco, USA) under a controlled culture environment. Penicillin (100U/mL) and streptomycin (100 mg/mL) were supplemented to the media.

### Transwell assay

Cell invasion was evaluated using Matrigel Invasion Chamber (8 μm) (Corning, shanghai, China) coated with 40 μL Extracellular Matrices (Sigma, USA) as per the manuals's recommendations. The procedure was outlined. In the transwell's upper chamber, 5 × 10^4^ cells were seeded (Sigma, USA). In the lower chamber, 30% FBS was added. Following 24 h, the cells adhering to the top layer were carefully collected. Crystal violet (0.1%) was used to stain the cells at the chamber's base. Finally, the invaded cells were quantified via the Image J software.

### Scratch assay (wound closure)

Cell migration was observed by scratch assay. Cells (1 × 10^6^ cells/well) were cultured in a 6-well plate a day in advance. A 10 μL pipette tip was utilized to make the wounds. After 24 h later, the photographs were taken, and the gap closure was estimated using the Image J software.

### Colony formation assay

Cells were trypsinized to form single-cell suspension. Approximately 1000 cells/well were allowed to grow in culture plates with complete media. Cells were fixed with 4% paraformaldehyde (PFA) after 2 weeks. The supernatant was removed and colonies were stained with crystal violet buffer for 20 min. Subsequently, the cell colonies were rinsed and dried naturally. Finally, the images of colonies were taken using the optical microscope. The number of colonies was detected using Image J software.

### Western blotting

A lysis buffer comprising proteinase inhibitors was used to extract total cellular protein. Western blotting was conducted by the established protocol. The antibodies listed below were used for this experiment: anti-SLC2A1 (Proteintech, 21829-1-AP), anti-β-actin (Proteintech, 20536-1-AP), anti-β-catenin (Cell Signaling Technology, D10A8), anti-METTL3 (ABclonal Technology, A19079), HK2 (Proteintech, 22029-1-AP), anti-LDHA (Proteintech, 19987-1-AP), YWHAZ (ABclonal Technology, A13370), anti-Histone H3 (Proteintech, 17168-1-AP), anti-Ubiquitin (Proteintech, 10201-2-AP), anti-β-TrCP (ABclonal Technology, A1656) and anti-c-Myc (Proteintech, 10828-1-AP).

### RNA stability assay

For this assay, actinomycin D (ActD, 5 μg/mL), an inhibitor of RNA polymerase, was employed for 0, 3, and 6 h. Then, the total RNA was collected from the indicated groups via the RNAiso Plus (TaKaRa Bio, Japan) reagent, followed by qRT-PCR analysis. The gene expression results were normalized to the β-actin (control gene) and calculated using the 2^-△△Ct^ method. The half-life of mRNA was determined via the following formula:

ln(C/C_0_) = -K_decay_t

K_decay_ is a constant of mRNA decay; The letter t is the inhibition time; C_0_ is the RNA concentration at 0 h; C is the RNA concentration at t hour.

The mRNA half-time (C_0_/C = 0.5) was calculated using the below equation:

t_1/2_ = In2/K_decay_

### Total RNA m6A quantification

EpiQuik m6A RNA Methylation Quantification Kit (Epigentek Group Inc., USA) was utilized to analyze the total RNA m6A level as per the manufacturers' specifications. The m6A levels were detected colorimetrically at 450 nm. The calculations were executed in line with the m6A standard curve.

### MeRIP-qPCR

A methylated RNA immunoprecipitation-qPCR (MeRIP-qPCR) assay was carried out to evaluate m6A modifications of RNAs. Briefly, RNA was collected via the RNAiso Plus (TaKaRa Bio, Japan) reagent. Then, poly(A)+ RNAs were purified by the VAHTSTM mRNA Capture Beads (Vazyme, China). Purified RNA was incubated with m6A antibody-beads compounds in an incubation buffer containing RNase inhibitor (Beyotime Institute of Biotechnology, China) at 4 °C. The enriched m6A RNAs were eluted and purified. The m6A enrichment levels of RNAs were analyzed by RT-PCR.

### RNA fluorescence *in situ* hybridization (RNA-FISH) assay

The FISH Tag™ RNA Multicolor Kit (Invitrogen, USA) and MAXIscript® Kit (Ambion, USA) were used to perform RNA-FISH assay as per the manual's guidelines. The cell samples were fixed using formaldehyde and permeabilized using Triton X-100. Then the hybridization was conducted using biotin-labeled RNA probes for SLC2A1-DT at 42°C overnight. After incubation with DAPI to define the nucleus, the cells were treated with antifade reagent under coverslips. The fluorescence intensities were measured using the Laser Scanning Confocal Microscope (LSCM).

### RNA-binding protein immunoprecipitation (RIP)

This assay was conducted with the RIP Kit (Magna RIP™, Millipore, USA) with specific antibodies. Briefly, RIP lysis buffer was added to the cells. The resultant cell lysate was kept with antibodies of interest (5 μg) and negative control antibody (IgG), respectively. RIP immunoprecipitation buffer was used to obtain the RNA-protein complexes. Following this, RNA purification was performed using the proteinase K buffer (150 μL) and Phenol-Chloroform-Isoamyl buffer and the co-precipitated RNAs were measured by qRT-PCR.

### Biotin-labeled RNA pull-down and mass spectrometry analysis

The biotin-labelled RNA pull-down assay was carried out in line with the guidelines provided by the manufacturer. Precisely, biotin-labelled RNA probes for SLC2A1-DT and truncates were designed and transcribed *in vitro* via specific primers comprising the T7 RNA-polymerase sequence. The Pierce RNA 3' End Desthiobiotinylation Kit (Thermo Scientific, USA) was utilized to label the 3' end of purified RNA products. The protein complexes pulled down by the SLC2A1-DT probe were obtained via the Pierce™ Magnetic RNA-Protein Pull-Down Kit (Thermo Scientific, USA). Then the beads containing the protein were washed and boiled for mass spectrometry analysis by Shanghai Applied Protein Technology Co. (Shanghai, China), or were subjected to a western blotting assay.

### Co-immunoprecipitation (Co-IP)

Cellular proteins were harvested according to the protein extraction steps, and the concentration of the extracted protein was adjusted to about 1 μg/μL. The agarose protein A bead suspension was mixed with 1 mL protein solution at 60 μL volume and incubated at 37℃ for 50 min. The purpose of this procedure was to improve experimental specificity. 600 μL of protein solution was removed, and the antibody against the target protein (1 μg) was added, followed by incubation at 4℃ for 12 h. Then 70 μL agarose protein A beads were added, and the reaction was performed at 4℃ for at least 2 h with light shock. The sample was centrifuged at the end, leaving the bottom bead to hold the protein-antibody complex onto the bead. The beads were washed with RIPA lysate buffer, followed by resuspension in 50 μL protein upwelling buffer. The samples were boiled for 10 min to enable denaturation before they were used for subsequent analysis.

### Lactate production assay

A lactic acid assay kit (Jiancheng, China) was used to detect lactate production by cells. Firstly, cancer cells were seeded into the 12-well plates (1 x 10^5^ cells/well), followed by transfection with the indicated control vectors or siRNA for 48 h. To detect the relative concentration of lactate, the standard curve of lactate was constructed. Finally, the absorbance of lactate in the culture media was examined by an ELISA plate reader (Thermo Fisher Scientific, USA) at 530 nm. The relative concentration of lactate production was normalized by the cell numbers and calculated using the standard curve.

### Glucose uptake assay

The fluorescence 2-NBDG assay kit (APExBIO, USA) was purchased for the measurement of glucose uptake. Cancer cells were allowed to grow into 12-well (1 x 10^5^ cells/well) plates, followed by transfection with siRNA or control vectors for 48 h. The culture media was changed by the Krebs Ringer Bicarbonate (KRB) buffer comprising 2-NBDG (100 µM) for 0.5 h at 37°C. KRB was used to wash the cells. The fluorescence intensity of cells was detected by flow cytometry. The protein concentration was used to normalize the fluorescence intensity.

### Cell proliferation assay

The propagation of tumor cells was quantified via the MTT assay. Cells were inoculated into culture 96-well plates and then monitored for 5 days. After 4 h incubation at 37°C with 20 μL MTT (5 mg/mL) in each well, 150 μL of DMSO (Sigma) was added. Finally, the absorbance was observed using the ELISA reader at 570 nm.

### Chromatin immunoprecipitation (ChIP)

The ChIP assay was conducted via an EZ-ChIPTM ChIP reagent (Millipore, MA, USA). Cells were plated in 15 cm plates, and cross-linked with 1% formaldehyde for 20 min. Following this, the cell lysate was ultrasonically sonicated to obtain the precise length of DNA (300-500 bp). The lysate was kept with a respective antibody to generate DNA-protein complexes. After the washing procedure, the DNA fragments were evaluated by qRT-PCR. Rabbit-IgG antibody (Proteintech, 30000-0-AP) was used as the control group.

### Immunohistochemistry (IHC)

This experiment was conducted as per the manufacturer's instructions. Firstly, formalin-fixed tissues were embedded with paraffin and then sectioned (4 µm) for the subsequent procedures. In brief, the tissues were mixed with EDTA antigen retrieval solution and incubated with the respective primary antibodies in a darkroom at 4℃ for 24 h. DAB was used for visualizing the immune complexes. Then hematoxylin was used for counterstaining the nucleus. Finally, protein expression was quantified by measuring the intensity and extent of staining at 200x magnification under a light microscope.

### Immunofluorescence

Tumor cells were allowed to grow on coated coverslips and kept with 4% PFA for 30 min, and 0.5% TritonX-100 for 15 min. Following a 30-min incubation with 5% Bovine serum albumin (Beyotime, China), the cells were incubated at 37°C for 2 h with respective primary antibodies (1:100). The Cy3-linked goat anti-rabbit IgG (Beyotime, China) was utilized as a secondary antibody. For staining of cell nuclei, DAPI (Beyotime, China) was incubated with cells for 5 min. Finally, the immunofluorescence images were taken by Zeiss LSM510 microscope (Germany).

### Luciferase activity assay

To evaluate the efficiency of the METTL3 promoter, cells were transfected using a pGL3 vector that contained either wild-type or mutant promoter along with a firefly luciferase plasmid. As an internal control, the plasmid pRL-TK was used. The luciferase activity assay was conducted using a Promega dual-luciferase reporter system. The activity of firefly luciferase was normalized to that of Renilla luciferase. To explore the role of SLC2A1-DT on the transcriptional activity of β-catenin, the FOP-FLASH and TOP-FLASH reporter plasmids were purchased from Beyotime Co. (China). The TOP-FLASH/FOP-FLASH ratio indicated the relative level of β-catenin promoter activity.

### Bioinformatics analysis

The clinical and transcriptome profiling data of patients with liver cancer has been retrieved from the TCGA database (https://portal.gdc.cancer.gov). To further understand the biological pathways associated with SLC2A1-DT, the gene set enrichment analysis (GSEA) software (http://www.broadinstitute.org/gsea) and Metascape (http://metascape.org) were utilized. ChIP-seq data was obtained from the Cistrome Data Browser (http://cistrome.org/db/#/). The R software (https://cran.r-project.org) was used to investigate the ATAC-seq data from the TCGA Publication Page (https://gdc.cancer.gov/about-data/publications/ATACseq-AWG), which was derived from 410 tissues from the TCGA dataset of primary human tumors.

### RNA isolation, reverse transcription, and quantitative real-time PCR (qRT-PCR)

RNAiso Plus reagent (TaKaRa Bio, Japan) was used to extract total RNA. The reverse transcription process was carried out using the TaKaRa Bio PrimeScript RT Master Mix Perfect Real Time kit. Furthermore, qRT-PCR analysis was conducted using the Premix Ex Taq II (TaKaRa Bio, Japan) and StepOnePlus real-time PCR system (Applied Biosystems, USA). By normalizing the corresponding transcript levels of β-actin, the 2^-△△Ct^ method was applied. Table S1 provides a list of the primer sequences.

### Transfection

SLC2A1-DT siRNA(siSLC2A1-DT), YWHAZ siRNA (siYWHAZ), β-catenin siRNA (siβ-catenin), METTL3 siRNA (siMETTL3), YTHDF1 siRNA (siYTHDF1), siRNA c-Myc (sic-Myc), and negative control siRNAs (siNC) were procured from GenePharma Co. (Suzhou, China). SLC2A1-DT, YTHDF1, β-catenin, METTL3, YTHDF1, c-Myc, control vector and corresponding truncated plasmids were purchased from Genechem Co. (Shanghai, China). Hiperfect (QIAGEN), Lipofectamine 2000 (Invitrogen, USA), and Opti-MEM I (Invitrogen, USA) were utilized for the transfection of siRNAs and plasmids. For stable transfections, the sequence of SLC2A1-DT siRNA and β-catenin were constructed into the lentiviral vectors, respectively. The control group consisted of empty lentiviral vectors. The vectors and siRNA sequences are presented in Table S2.

### Xenograft studies

The SMMC-7721 cells were transfected with lentivirus carrying either a negative control (LV-NC) or SLC2A1-DT sequence (LV-SLC2A1-DT). From HFK Bio-Technology Co. (Beijing, China), male BALB/c nude mice (4 weeks old) were acquired. Injections of SMMC-7721 cells were administered to the dorsal flanks or tail veins of nude mice. After every six days, the mentioned formula V = 0.5L (length) × W2 (width) was applied to quantify the change in tumor volume. All mice were sacrificed, and their respective tumors were measured after 4 weeks. All animal-related experiments received approval from the Animal Research Committee of the Academic Medical Center at Huazhong University of Science and Technology (Wuhan, China). Each procedure adhered to the recommended rules for the care and use of animals.

### Statistical analyses

Data was displayed as means ± standard deviation (SD). Two groups were compared via the Student's t-test. To identify the association between gene expression within the groups, Pearson correlation analysis was carried out. Meanwhile, the SLC2A1-DT expression and the clinical characteristics were analyzed by χ2 tests. To compare the variance between survival and hazard ratios (HR), the log-rank test and Cox regression models were implemented. The significance level was identified for values of. Significant values were defined as * p ≤ 0.05, ** *p* ≤ 0.01; and *** *p* ≤ 0.001, respectively.

## Results

### Enhanced SLC2A1-DT level in HCC and linked with poor prognosis

**Figure 1:**
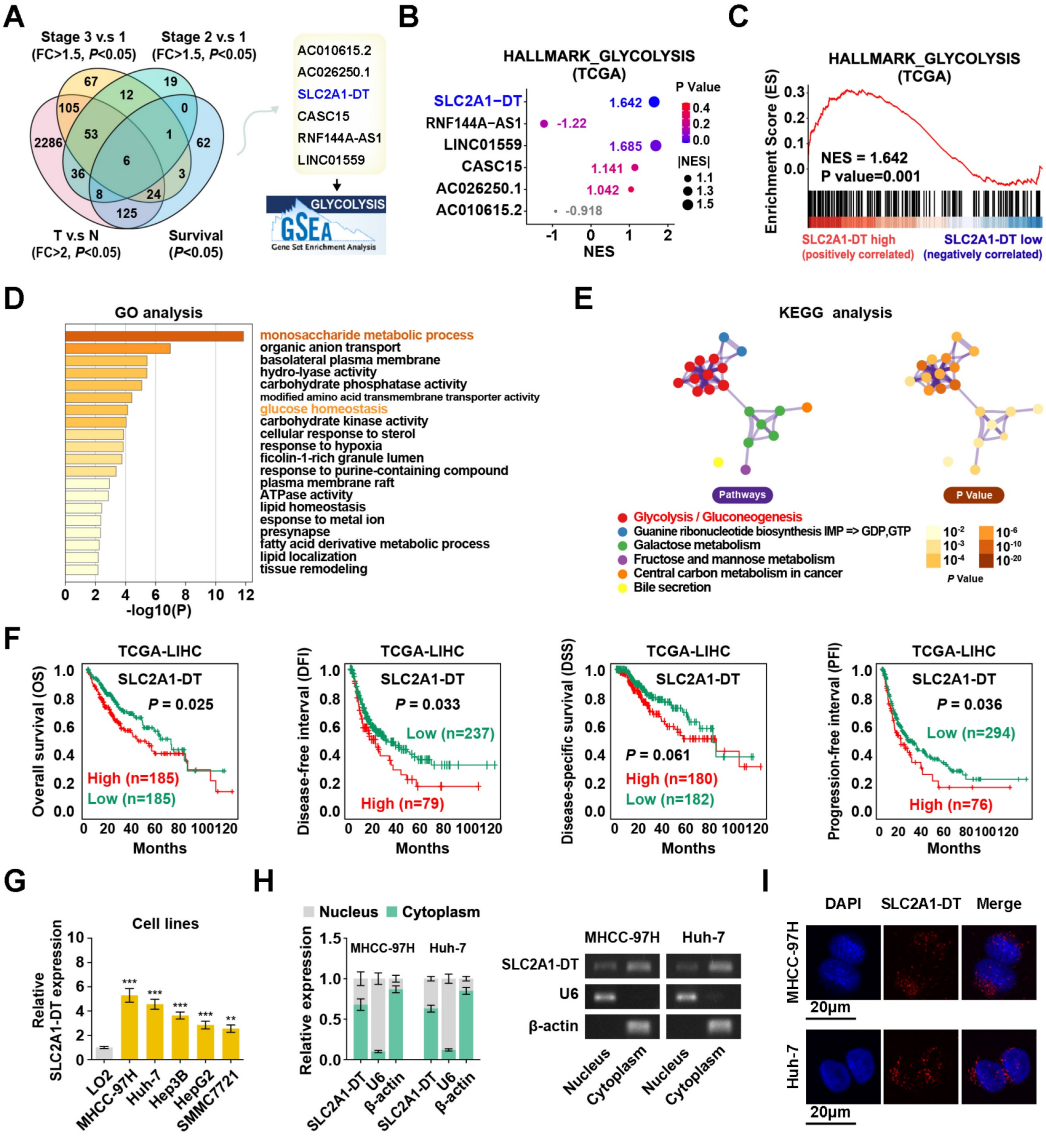
** Enhanced SLC2A1-DT expression in HCC associated with poor prognosis. (A)** Venn diagram (left panel) showing the differentially expressed lncRNAs related to poor outcomes in the LIHC cohort from the TCGA database. The illustration (right panel) depicts the lncRNAs associated with glycolysis. **(B)** A bubble graph illustrating the normalized enrichment score (NES) and the GSEA of alternative lncRNAs using the glycolysis gene set. **(C)** GSEA enrichment plot indicating the enrichment of SLC2A1-DT in the gene set of hallmark glycolysis genes. **(D-E)** The GO and KEGG analyses of genes associated with SLC2A1-DT (|r| > 0.3, P < 0.05) in the TCGA-LIHC dataset. **(F)** Kaplan-Meier analyses of overall survival (OS) (cutoff value =4.31), disease-free interval (DFI) (cutoff value =9.37), disease-specific survival (DSS) (cutoff value =4.30), and progression-free interval (PFI) (cutoff value =10.63) in the LIHC cohort with low and high SLC2A1-DT levels via the log-rank test. **(G)** The relative level of SLC2A1-DT in 5 HCC cell lines (MHCC-97H, Huh-7, Hep3B, HepG2, SMMC-7721) and normal hepatocyte cells (LO2). **(H)** qRT-PCR (left panel) revealing the nuclear and cytoplasmic distribution of SLC2A1-DT in MHCC-97H and Huh-7 cells. In the right panel, β-actin and U6 were employed as cytoplasmic and nuclear markers, respectively, while the PCR products were confirmed via 2% agarose gel. **(I)** Confocal microscopy showing RNA-FISH results using Alexa Fluor® 594-labeled probe (red). All nuclei were visualized via DAPI expression (blue). The data are reported as the mean ± SD of three independent/separate experiments. Scale bar: 20 μm.

To determine potential glycolysis-related lncRNAs associated with HCC tumorigenesis, we examined the differentially expressed lncRNAs in HCC tissues from the TCGA database (tumor vs normal; fold change > 2, *P* < 0.05). Results displayed that 6 lncRNAs were associated with prognosis (*P* < 0.05) in HCC patients with different clinical stages (stage 3 vs stage 1, stage 2 vs stage 1; fold change > 1.5; *P* < 0.05) (Fig. 1A). Among these lncRNAs, SLC2A1-DT was found to be most significantly correlated with the glycolysis pathway in the gene set enrichment analysis (GSEA), making it our primary focus (Fig. 1B-C). Gene Ontology (GO) and Kyoto Encyclopedia of Genes and Genomes (KEGG) analyses showed a correlation between SLC2A1-DT and monosaccharide metabolic process, glucose homeostasis, and glycolysis/gluconeogenesis (Fig. 1D-E). The TCGA database indicated that SLC2A1-DT was upregulated in HCC patients with advanced clinical stages and most types of tumors (Fig. S1A-B). Survival analyses suggested that high expression of SLC2A1-DT was linked with shorter overall survival (OS), disease-free interval (DFI), disease-specific survival (DSS), and progression-free interval (PFI) of HCC patients (Fig. 1F). Subsequently, the link between SLC2A1-DT level and different clinicopathological characteristics in the TCGA-LIHC dataset was analyzed. We observed that SLC2A1-DT expression was substantially related to the TNM stages, T stages, and G grades (Fig. S1C, Table S3). Univariate/multivariate regression analyses exhibited that the expression level of SLC2A1-DT was a distinct predictor of the clinical outcomes of HCC patients with a significant hazard rate (HR) (Table S4).

Moreover, SLC2A1-DT expression was significantly elevated in HCC cells (MHCC-97H, Huh-7, Hep3B, HepG2 and SMMC7721) than in normal human hepatic cells (LO2) (Fig. 1G). Subcellular fractionation assays suggested that SLC2A1-DT was mainly located in the cytoplasm (Fig. 1H). The FISH assays showed similar results (Fig. 1I). Furthermore, the secondary structure and the low protein-coding ability of SLC2A1-DT were predicted by online web servers, respectively (Fig. S1D-G). Therefore, SLC2A1-DT was found to be overexpressed in HCC tissues, suggesting its role as an oncogenic factor in HCC progression.

### SLC2A1-DT promotes HCC cell propagation, invasion, and metastasis by enhancing glycolysis

**Figure 2:**
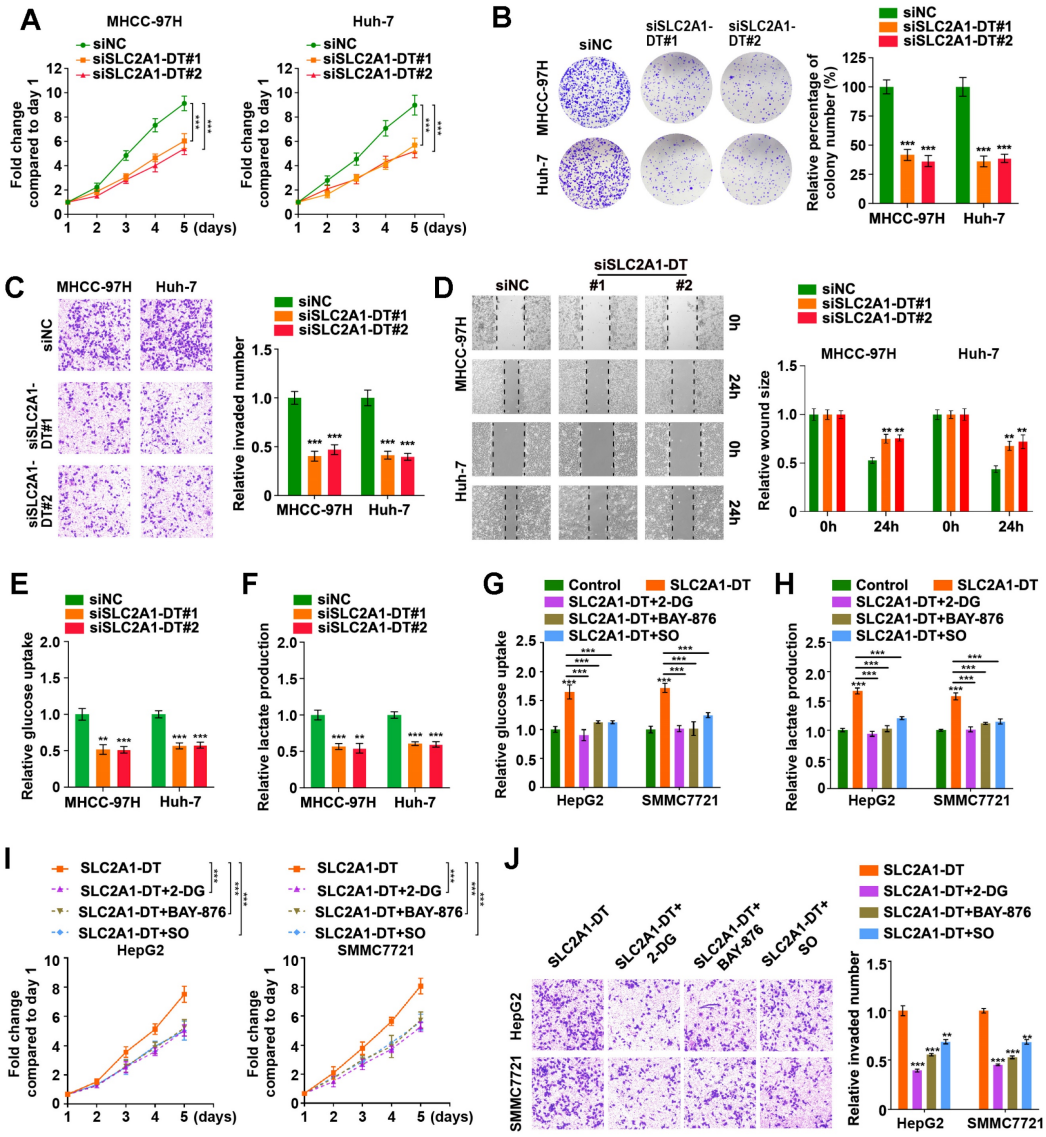
** SLC2A1-DT promotes propagation, invasion, and metastasis of HCC cells by enhancing glycolysis. (A)** MTT colorimetric assay showing the knockdown impact of SLC2A1-DT on the proliferative rate of MHCC-97H and Huh-7 cells. **(B)** Colony formation assays (left panel) and relative quantification (right) indicating the effects of SLC2A1-DT knockdown on the colony formation ability of MHCC-97H and Huh-7 cells. **(C)** Transwell assay (left) and relative measurements (right) assessed the invasive capability of MHCC-97H and Huh-7 cells after being transfected with siSLC2A1-DT. **(D)** Wound-healing assay showing the effect of SLC1A1-DT silencing on the migration of MHCC-97H and Huh-7 cells. Representative images (left panel) showing the relative wound sizes (right panel). **(E-F)** The levels of glucose uptake and lactate production in MHCC-97H and Huh-7 cells transfected with siNC or siSLC2A1-DT were measured. **(G-H)** Measurement of glucose uptake and lactate production levels in HepG2 and SMMC7721 cells with SLC2A1-DT augmentation and 2-DG (2mM), BAY-876 (2 μM) or Sodium oxamate (20mM) treatment. **(I-J)** MTT colorimetric assay and transwell assay showing the proliferative and invasive potential of HepG2 and SMMC7721 cells with SLC2A1-DT upregulation and 2-DG (2 mM), BAY-876 (2 μM) or Sodium oxamate (20 mM) treatment.

To evaluate the precise role of SLC2A1-DT in HCC, we used siRNA to knockdown SLC2A1-DT expression (siSLC2A1-DT#1 and siSLC2A1-DT#2) in MHCC-97H and Huh-7 cells, which had a higher expression of SLC2A1-DT (Fig. S2A). After the knockdown of SLC2A1-DT, the proliferation, invasion, and migration of the above HCC cells were remarkably reduced (Fig. 2A-D). Furthermore, the knockdown of SLC2A1-DT significantly decreased glucose uptake and lactate production in MHCC-97H/Huh-7 cells (Fig. 2E-F). In contrast, transfection with pcDNA-SLC2A1-DT overexpression plasmid significantly increased the propagation, invasion, and migration in HepG2 and SMMC7721 cells, which had a lower expression of SLC2A1-DT (Fig. S2B-F). Moreover, SLC2A1-DT overexpression increased the glycolysis in HepG2 and SMMC7721 cells, which was inhibited by treatment with 2-DG (inhibitor of glycolysis), BAY-876 (inhibitor for GLUT1) or Sodium oxamate (SO, inhibitor for LDHA) (Fig. 2G-H). However, these inhibitors did not influence the expression of SLC2A1-DT (Fig. S2G). Consistently, the cells with SLC2A1-DT overexpression and treatment with 2-DG, BAY-876 or Sodium oxamate exhibited decreased ability for propagation and metastasis (Fig. 2I-J). Therefore, the results above implied that SLC2A1-DT enhanced the propagation, invasion, and migration of HCC cells in a glycolysis dependent manner.

### SLC2A1-DT interacts with YWHAZ in HCC cells

**Figure 3:**
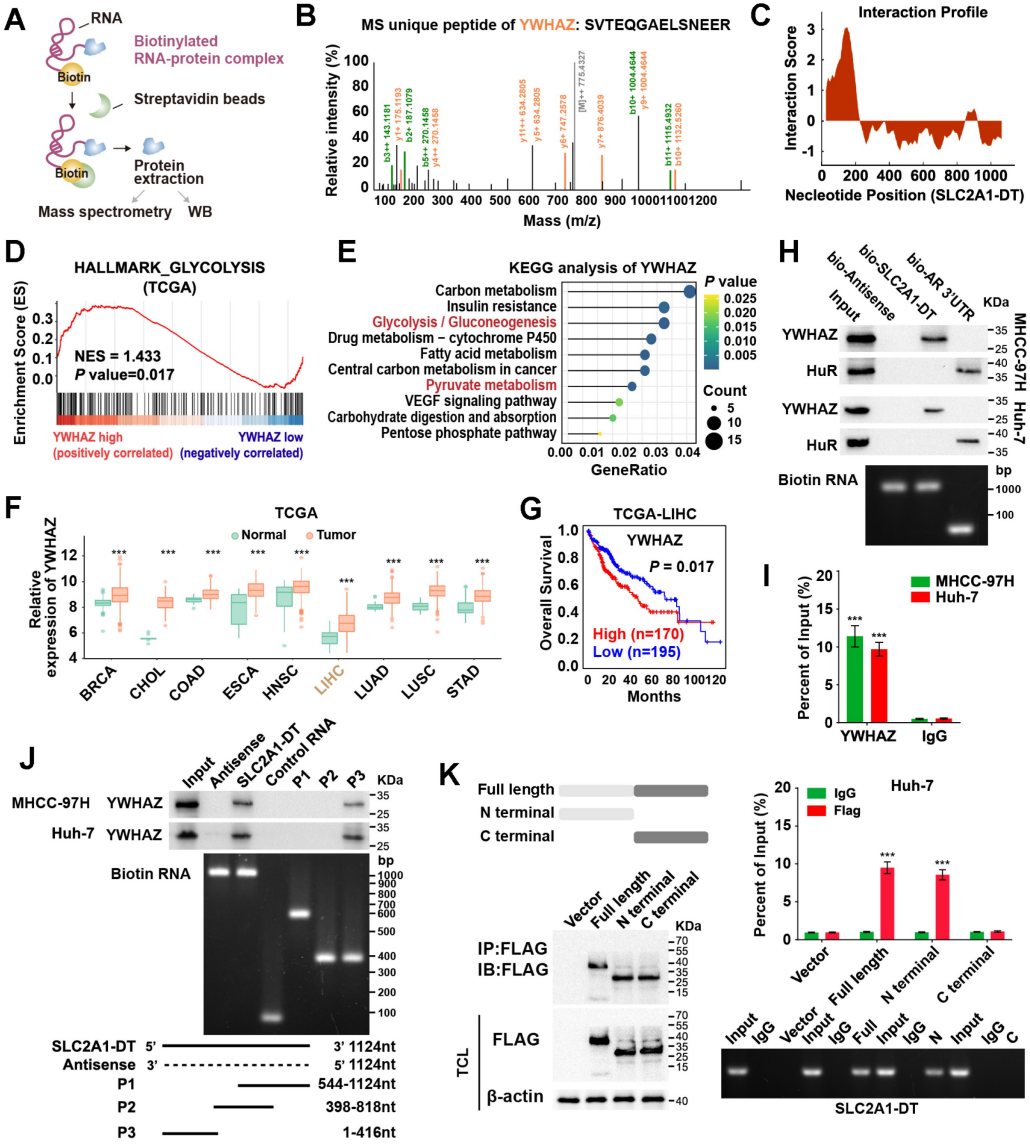
** SLC2A1-DT interacts with YWHAZ in HCC cells. (A)** An illustration showing the process of RNA pulldown followed by protein sample detection. SLC2A1-DT was labeled with biotin, and the beads were labeled with Streptavidin. **(B)** The connection between SLC2A1-DT and YWHAZ protein is verified by mass spectrometry (MS). **(C)** The possible binding sites between SLC2A1-DT and YWHAZ are evident in the RNA interaction profile. **(D)** GSEA analysis of YWHAZ-correlated genes derived from the TCGA-LIHC dataset. **(E)** KEGG analysis of YWHAZ-correlated genes. **(F)** The overall expression of YWHAZ in multiple human tumors from the TCGA database. **(G)** The log-rank test (cutoff value = 13264.27) was used to generate Kaplan-Meier curves depicting the survival of HCC patients in the TCGA-LIHC dataset at low and high expression levels of YWHAZ. **(H)** The proteins derived from biotinylated RNA pull-down assays were examined via western blotting using anti-YWHAZ and anti-HuR primary antibodies as a control, HuR-binding regions from RNA originating from the 3'UTR of the androgen receptor (AR) were used. Semi-quantitative RT-PCR was performed to confirm the presence of biotinylated RNA. **(I)** RIP assay was conducted using anti-YWHAZ antibody and anti-IgG antibody. Relative enrichment representing the expression levels of SLC2A1-DT to be associated with the expression of YWHAZ protein, relative to an input control after immunoprecipitation with the anti-YWHAZ antibody, compared with that of anti-IgG antibody. **(J)** RNA pulldown assay using anti-YWHAZ antibody indicating the interaction between a series of SLC2A1-DT truncates and YWHAZ protein. The presence of biotinylated RNA was measured by Semi-quantitative RT-PCR. **(K)** The RIP assay (lower) was used to determine the binding efficiencies of various YWHAZ protein fragments with SLC2A1-DT in Huh-7 cells. The schematic representations of the YWHAZ protein and its two truncated mutants were used in this study. The presence of SLC2A1-DT was measured by Semi-quantitative RT-PCR.

Given that SLC2A1-DT was mainly localized in the cytoplasm, we speculated that it might exert its function by interacting with other proteins. To identify the underlying protein interacting with SLC2A1-DT, we conducted RNA pull-down assay in MHCC-97H cells (Fig. 3A). Mass spectrometry analysis revealed that tyrosine 3-monooxygenase/tryptophan 5-monooxygenase activation protein zeta (YWHAZ) was the protein with unique peptides that was being pulled down by biotin-labelled SLC2A1-DT (Fig. 3B, Table S5). Meanwhile, the catRAPID algorithm revealed a strong interaction between SLC2A1-DT and YWHAZ (Fig. 3C). GSEA and KEGG analyses also showed that YWHAZ was associated with glycolysis and pyruvate metabolism, respectively (Fig. 3D-E). Data from the TCGA database suggested that YWHAZ was upregulated in various kinds of tumors in the TCGA database (Fig. 3F), and the high level of YWHAZ was related to poor outcomes in HCC patients (Fig. 3G). Western blot assay further indicated the presence of YWHAZ in the SLC2A1-DT RNA pull-down complex (Fig. 3H). RIP assay further validated the binding between SLC2A1-DT and YWHAZ (Fig. 3I). Moreover, deletion-mapping analyses demonstrated that the 1-416 nt region of SLC2A1-DT was necessary for its binding with YWHAZ (Fig. 3J). Cell culture binding assay revealed that the N terminal, but not the C terminal domain of Flag-tagged YWHAZ protein was essential for its binding with SLC2A1-DT (Fig. 3K). These findings revealed that SLC2A1-DT is specifically bound to YWHAZ in HCC cells.

### SLC2A1-DT/YWHAZ signaling increases the stability of β-catenin in HCC cells

A previously published study indicated that YWHAZ was related to β-catenin protein levels and suppressed its degradation 21. β-catenin is a multifunctional protein that plays a critical role in carcinogenesis and glycolysis through many downstream mechanisms 22. Thus, we further investigated whether SLC2A1-DT/YWHAZ signaling affected β-catenin expression in HCC cells. The outcomes revealed that the knockdown or high expression of YWHAZ reduced or elevated β-catenin protein levels, respectively, but the β-catenin mRNA level was not proportionally altered in HCC cells (Fig. S3A and B).

**Figure 4:**
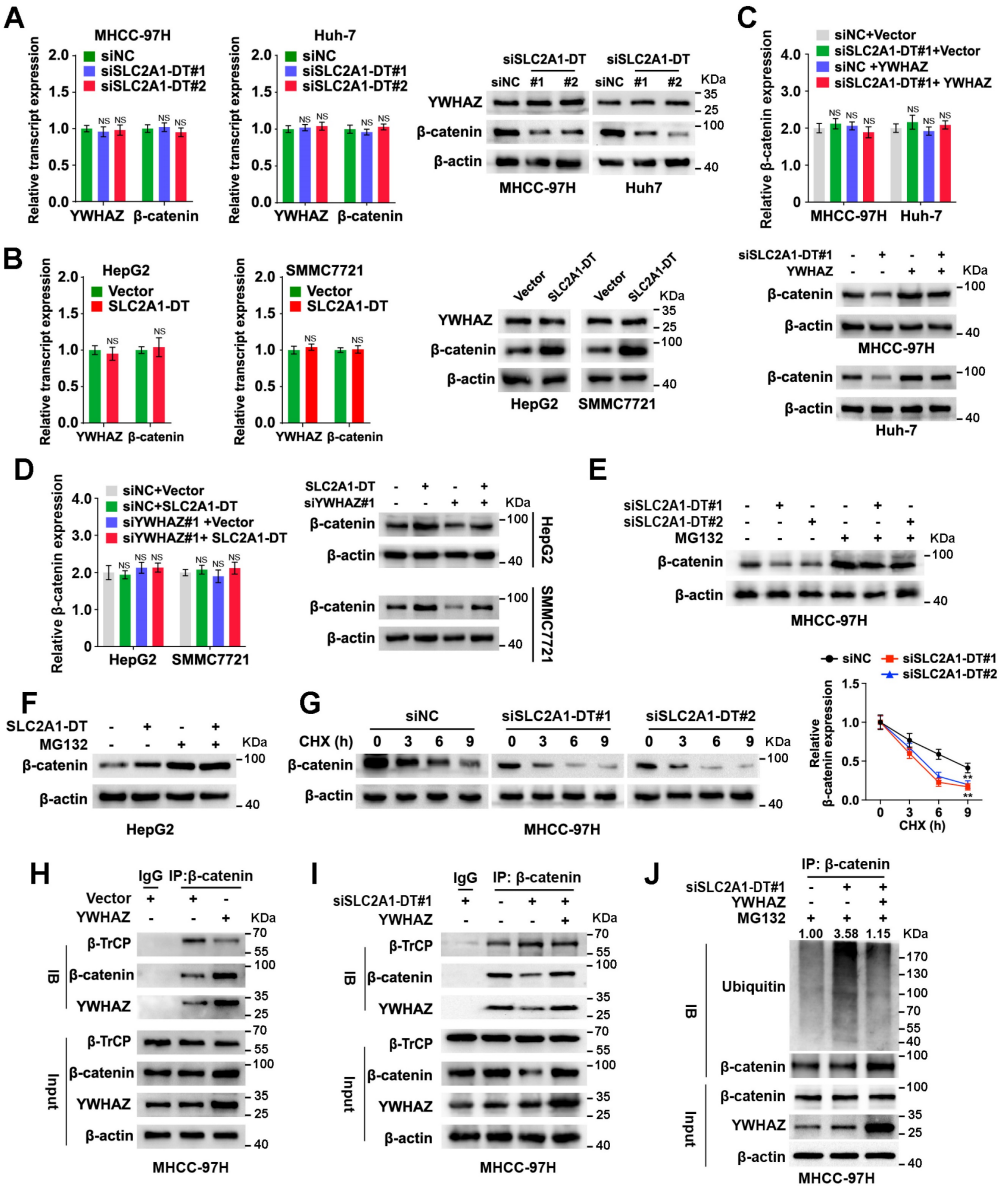
** SLC2A1-DT/YWHAZ signaling promotes the β-catenin stability in HCC cells. (A)** Western blot (right) and qRT-PCR (left) illustrate the protein and transcript levels of β-catenin and YWHAZ, respectively, in MHCC-97H and Huh-7 cells after transfection with siNC or siSLC2A1-DT. **(B)** The RNA and protein levels of YWHAZ and β-catenin were detected by qRT-PCR (left) and western blot (right) in HepG2 and SMMC7721 cells with or without SLC2A1-DT overexpression. **(C)** The levels of the β-catenin transcript and protein in MHCC-97H and Huh-7 cells were determined by qRT-PCR (upper) and western blot (lower) after siNC or siSLC2A1-DT transfection, as well as vector or YWHAZ plasmid co-transfection. **(D)** The protein and transcript levels of β-catenin in HepG2 and SMMC7721 cells were determined by qRT-PCR (left) and western blot (right) after transfection with vector or SLC2A1-DT plasmids and co-transfection with siNC or siYWHAZ plasmids. **(E)** MHCC-97H cells were transfected with siSLC2A1-DT for 48h followed by harvesting the cells for western blotting for β-catenin after treatment with or without MG132 (10 μmol/L). **(F)** HepG2 cells were transfected with SLC2A1-DT plasmids for 48h. Cell lysates were harvested for western blot analysis using anti-β-catenin antibody after treatment with or without MG132 (10 μmol/L). **(G)** MHCC-97H cells were transfected with siSLC2A1-DT for 48h followed by treatment with cycloheximide (CHX, 12.5 g/mL) and then collected for western blotting (left panel) at different time points. The relative intensity (right) of protein bands was measured using the Image J software. **(H)** The protein from YWHAZ expressing cells were immunoblotted with anti-β-catenin, anti-β-TrCP, and anti-YWHAZ antibodies. **(I)** YWHAZ plasmids were co-transfected with siSLC2A1-DT to generate MHCC-97H cell lysates, which were subsequently immunoprecipitated using anti-β-catenin, anti-β-TrCP, and anti-YWHAZ antibodies. **(J)** A combination of siSLC2A1-DT and YWHAZ plasmids was used to transfect MHCC-97H cells. Following a 48-hour treatment with MG132, cell lysates were collected for western blot assays. The protein lysates were immunoprecipitated with anti-β-catenin, anti-YWHAZ, and anti-ubiquitin antibodies.

Similarly, knockdown or overexpression of SLC2A1-DT reduced or induced the protein expression but not mRNA expression of β-catenin in HCC cells (Fig. 4A and B, Fig. S3C and D), which was remarkably rescued or reversed by ectopic expression and depletion of YWHAZ (Fig. 4C-D, Fig. S3E-H). Furthermore, the results showed that the proteasome inhibitor, MG132, distinctively attenuated the degradation of β-catenin protein which was induced by SLC2A1-DT silencing or overexpression, respectively (Fig. 4E and F, Fig. S3I and J). Meanwhile, SLC2A1-DT knockdown and overexpression shortened or prolonged the half-life of β-catenin protein, respectively, when HCC cells were treated with cycloheximide (CHX), a protein synthesis inhibitor (Fig. 4G, Fig. S3K and L).

A previously published study showed that YWHAZ suppressed the binding between E3 ligase β-TrCP and β-catenin, which inhibited the degradation of β-catenin 21. Thus, we wondered whether SLC2A1-DT/YWHAZ signaling promoted the disassociation between β-TrCP and β-catenin, which could stabilize β-catenin. As expected, the Co-IP assay suggested that YWHAZ overexpression facilitated the binding of YWHAZ and β-catenin, and repressed the binding between β-TrCP and β-catenin (Fig. 4H). Moreover, SLC2A1-DT knockdown visibly reduced the binding of YWHAZ with β-catenin but enhanced the binding of β-TrCP with β-catenin. Therefore, SLC2A1-DT knockdown induced the ubiquitination and degradation of β-catenin, which was rescued by YWHAZ overexpression (Fig. 4I and J, Fig. S3M). Conversely, SLC2A1-DT overexpression increased the binding between YWHAZ and β-catenin but decreased the binding of β-TrCP with β-catenin, therefore, inhibited the ubiquitination and degradation of β-catenin, which was reversed by the knockdown of YWHAZ (Fig. S3N-P). Importantly, the results showed that SLC2A1-DT overexpression caused β-catenin accumulation in the cytoplasm and nucleus of HepG2 and SMMC7721 cells (Fig. S3Q-S). Subsequently, we identified the effect of SLC2A1-DT on β-catenin transactivation. The knockdown or overexpression of SLC2A1-DT attenuated or facilitated the transcription of β-catenin in HCC cells which were co-transfected with the TOP/FTO-flash report vectors (Fig. S3T). Thus, the above data showed that SLC2A1-DT enhanced the YWHAZ-mediated stability of β-catenin.

### SLC2A1-DT/ β-catenin axis enhances glycolysis by regulating glycolytic genes

**Figure 5:**
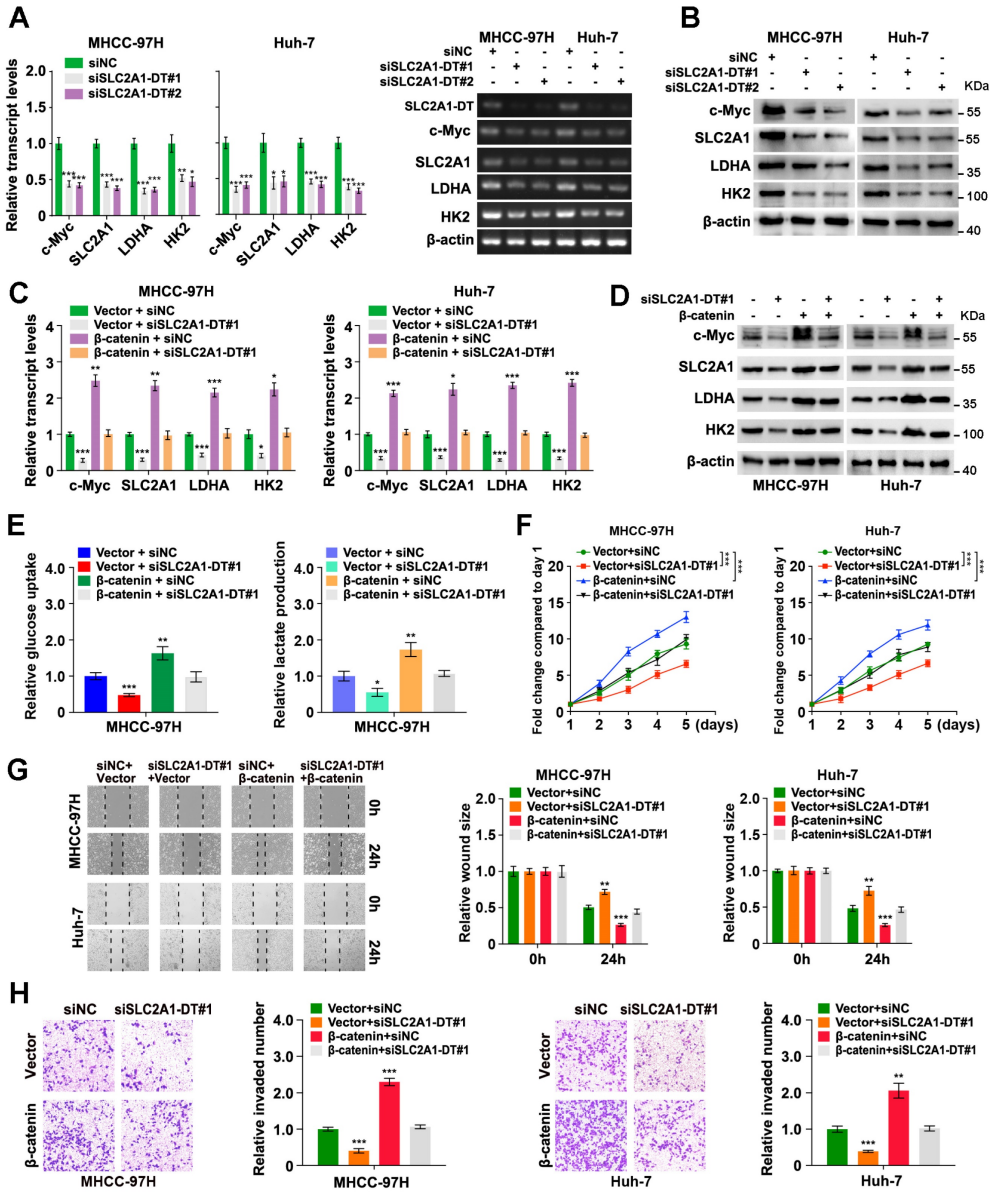
** SLC2A1-DT/ β-catenin enhances glycolysis by regulating glycolytic genes. (A-B)** The levels of mRNA (left) and protein (right) levels of c-Myc and downstream genes, including SLC2A1, LDHA, and HK2 in MHCC-97H cells transfected with siSLC2A1-DT. **(C-D)** Detection of the mRNA (left) and protein levels (right) of c-Myc and downstream genes, including SLC2A1, LDHA, and HK2, after transfection of MHCC-97H cells with siSLC2A1-DT, or co-transfection with β-catenin. **(E)** Detection of glucose uptake (left) and the lactate secretion levels (right) after transfection of MHCC-97H cells with vector or β-catenin plasmids, and co-transfection with siSLC2A1-DT. **(F-H)** The potential of HCC cells for proliferation, migration, and invasion was evaluated via MTT, wound healing, and transwell assays, respectively, after transfection with siNC or siSLC2A1-DT, and co-transfection with vector or β-catenin plasmids.

Since the above findings revealed the role of SLC2A1-DT in increasing the stability of and transactivation of β-catenin, we evaluated the effect of SLC2A1-DT on the expression of β-catenin target genes. The results revealed that SLC2A1-DT silencing or overexpression attenuated or facilitated the expression of c-Myc and glycolytic genes (SLC2A1, LDHA, and HK2), the downstream gene of β-catenin 23, 24 (Fig. 5A and B, Fig. S4A), respectively. Importantly, rescue experiments revealed that upregulation or depletion of β-catenin rescued the change in the expression of c-Myc, SLC2A1, LDHA, and HK2 stimulated by the silencing or overexpression of SLC2A1-DT, respectively (Fig. 5C and D, Fig. S4B-E). Meanwhile, overexpression or knockdown of β-catenin reversed the SLC2A1-DT mediated effects on the glucose uptake, lactate production, propagation, invasion, and metastasis of HCC cells (Fig. 5E-H, Fig. S4F-I). The above results suggested that SLC2A1-DT enhanced the glycolytic rate, propagation, migration and invasion of HCC cells by modulating β-catenin-dependent glycolytic genes.

### METTL3-mediated m6A modification controls the stability of SLC2A1-DT

**Figure 6:**
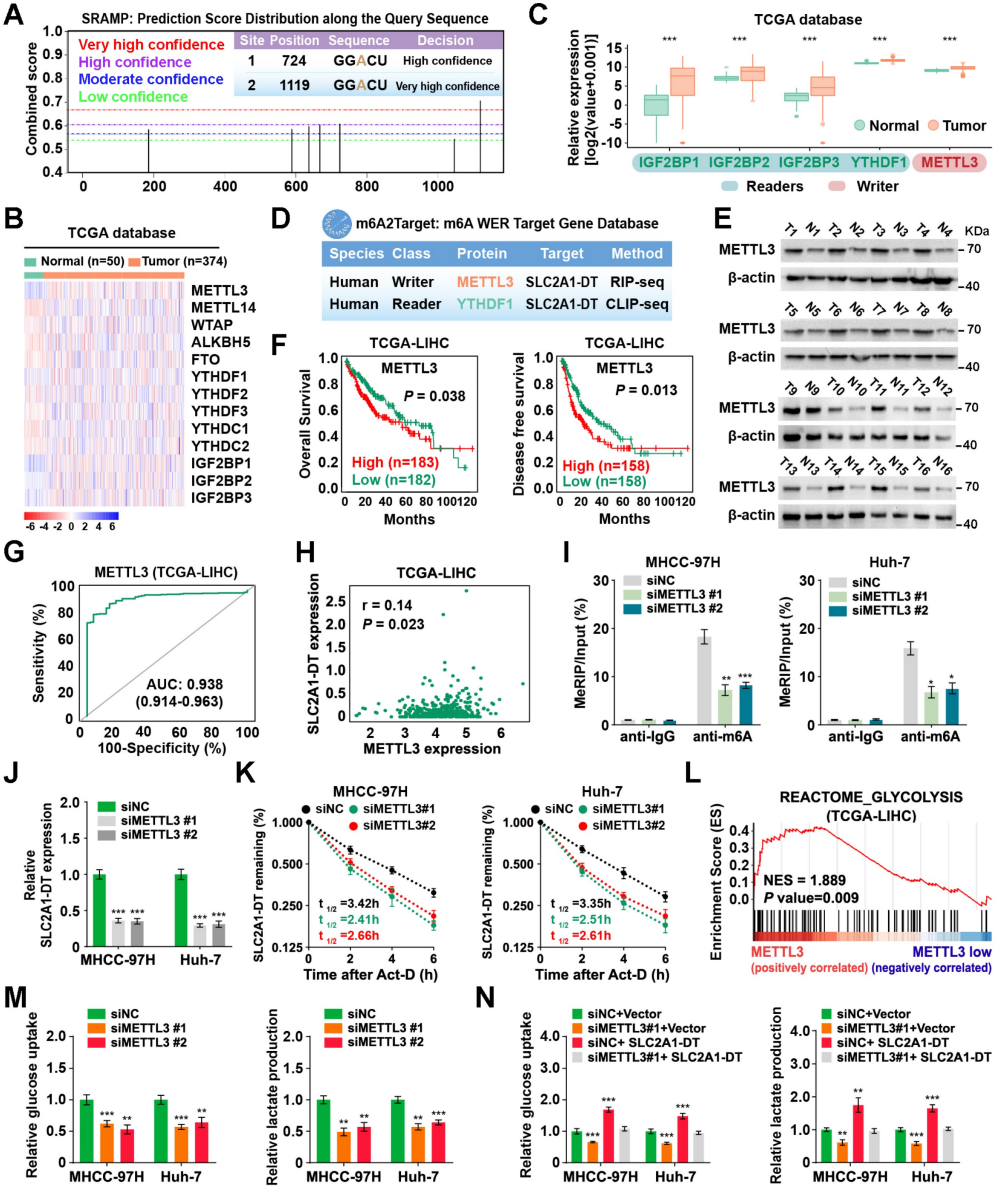
** METTL3-mediated m6A modification controls the stability of SLC2A1-DT. (A)** The potential m6A binding sites of SLC2A1-DT using the online tool SRAMP (http://www.cuilab.cn/sramp/). **(B-C)** Heatmap and boxplot profiling of the expression of m6A regulatory genes using the TCGA-LIHC dataset. **(D)** Identification of SLC2A1-DT as the potential target of METTL3 and YTHDF1 using the m6A2Target database (http://m6a2target.canceromics.org/#/). **(E)** The protein levels of METTL3 in HCC tissues and adjacent non-malignant tissues. T: Tumor tissue; N: Normal tissue. **(F)** Kaplan-Meier analyses of OS and DFI in TCGA-LIHC dataset with low and high METTL3 levels. **(G)** The ROC curve of METTL3 (AUC = 0.938, 95% CI: 0.914 to 0.963) in the TCGA-LIHC dataset. **(H)** The correlation analysis between METTL3 and SLC2A1-DT in the TCGA-LIHC dataset from the GEPIA database (http://gepia.cancer-pku.cn/index.html). (**I**) The MeRIP-qPCR was used to determine whether METTL3 silencing affected the levels of m6A with SLC2A1-DT. **(J)** The levels of SLC2A1-DT were evaluated by qRT-PCR in HCC cells after METTL3 silencing. **(K)** The stability of SLC2A1-DT RNA was measured in HCC cells after METTL3 silencing. **(L)** GSEA analysis for METTL3 by gene set of reactome_glycolysis in the TCGA-LIHC dataset. **(M)** The relative levels of glucose uptake (left panel) and lactic acid levels in media (right panel) were measured in HCC cells transfected with siNC or siMETTL3. **(N)** The MHCC-97H and Huh-7 cells, which were transfected with siNC or siMETTL3, as well as those co-transfected with vector or SLC2A1-DT, were used to assess the respective levels of glucose uptake (left) and lactic acid in the media (right).

Previous research has reported the mode of action of m6A modification in modulating RNA stability 25. Interestingly, we revealed 2 GGACU m6A motifs in the SLC2A1-DT sequence through the online bioinformatics tools SRAMP (http://www.cuilab.cn/sramp) (Fig. 6A). Therefore, we sought to identify whether the modification of m6A was responsible for the up-regulation of SLC2A1-DT in HCC. Meanwhile, analysis from the TCGA database displayed that m6A-related genes were upregulated in HCC tissues (Fig. 6B-C). The m6A2Target database (http://m6a2target.canceromics.org/#/) identified SLC2A1-DT as a potential target of METTL3 and YTHDF1 (Fig. 6D). Specifically, TCGA analysis showed that METTL3 was upregulated in numerous tumors, and the upregulation was validated by western blot assays from HCC tissues versus normal hepatic tissues (Fig. 6E, Fig. S5A). Moreover, the m6A content was elevated in HCC tissues compared with normal tissues (Fig. S5B). Meanwhile, the OS and DFS curves from the TCGA dataset represented that patients with high METTL3 levels had poor prognosis (Fig. 6F). The receiver operating characteristic (ROC) curves suggested that METTL3 was a sensitive diagnostic marker in HCC (Fig. 6G). Moreover, METTL3 was positively correlated with SLC2A1-DT in the TCGA database (Fig. 6H). Subsequently, mRNA and protein expression of METTL3 in HCC cell lines were also upregulated compared with normal liver cells (Fig. S5C). These results inspired us to explore whether METTL3-mediated m6A modification regulated the expression of SLC2A1-DT.

To identify the m6A database results, we conducted a MeRIP-qPCR assay to detect the m6A modifications of SLC2A1-DT. The findings revealed that m6A modification was abundantly present in SLC2A1-DT (Fig. 6I). In addition, the m6A enrichment and SLC2A1-DT levels were decreased or increased in HCC cells subjected to METTL3 inhibition or upregulation (Fig. 6I-J, Fig. S5D-F), respectively. Moreover, RNA stability assays showed that the inhibition or upregulation of METTL3 decreased or enhanced the RNA stability of SLC2A1-DT (Fig. 6K, Fig. S5G), respectively.

To further evaluate METTL3's biological function in HCC cells, we performed GSEA analysis, and the outcomes exhibit that METTL3 was strongly linked with glycolysis (Fig. 6L). Indeed, depletion or upregulation of METTL3 remarkably reversed or rescued glucose uptake and lactate production in HCC cells, which were stimulated by SLC2A1-DT overexpression or knockdown (Fig. 6M-N, Fig. S5H and I), respectively. Collectively, these results implied that METTL3 increased SLC2A1-DT stability in a m6A-dependent manner.

### YTHDF1 is required for m6A mediated stabilization of SLC2A1-DT

Since previous studies suggested that YTH N6-Methyladenosine RNA binding protein 1 modulated the stability of m6A-modified RNAs catalyzed by METTL3 26, 27, we further evaluated whether YTHDF1 regulated the m6A modification mediated stability of SLC2A1-DT. The TCGA analysis revealed that YTHDF1 was upregulated in various types of tumors, and was related to poor prognosis of LIHC patients (Fig. S6A and B). ROC curves analysis also indicated that YTHDF1 expression had a high value for predicting the survival of LIHC (Fig. S6C). Data from the ENCORI database suggested that the YTHDF1 level was substantially related to SLC2A1-DT expression in LIHC (Fig. S6D). These outcomes inspired us to explore further whether YTHDF1 was involved in the regulation of m6A modification of SLC2A1-DT by METTL3. Coincidently, the biotinylated RNA pull-down assay showed that YTHDF1 is specifically bound to SLC2A1-DT (Fig. S6E). Meanwhile, RIP assay with YTHDF1 antibody further confirmed an association between YTHDF1 and SLC2A1-DT (Fig. S6F). RIP analysis results revealed that METTL3 silencing or overexpression substantially reduced or increased the binding of YTHDF1 to SLC2A1-DT (Fig. S6G and H). Moreover, SLC2A1-DT expression was rescued or reversed by YTHDF1 upregulation or downregulation in METTL3 depleted or overexpressing HCC cell lines (Fig. S6I-K). Of note, YTHDF1 downregulation significantly decreased the RNA stability of SLC2A1-DT RNA (Fig. S6L). Additionally, decreased or increased glucose uptake and lactate production in METTL3 depleted or overexpressing cells were rescued or reversed by YTHDF1 overexpression or depletion (Fig. S6M-P), respectively. Collectively, YTHDF1 enhanced the stability of SLC2A1-DT through the recognition of m6A modification catalyzed by METTL3.

### METTL3 expression is reciprocally regulated by SLC2A1-DT/c-Myc pathway in HCC

**Figure 7:**
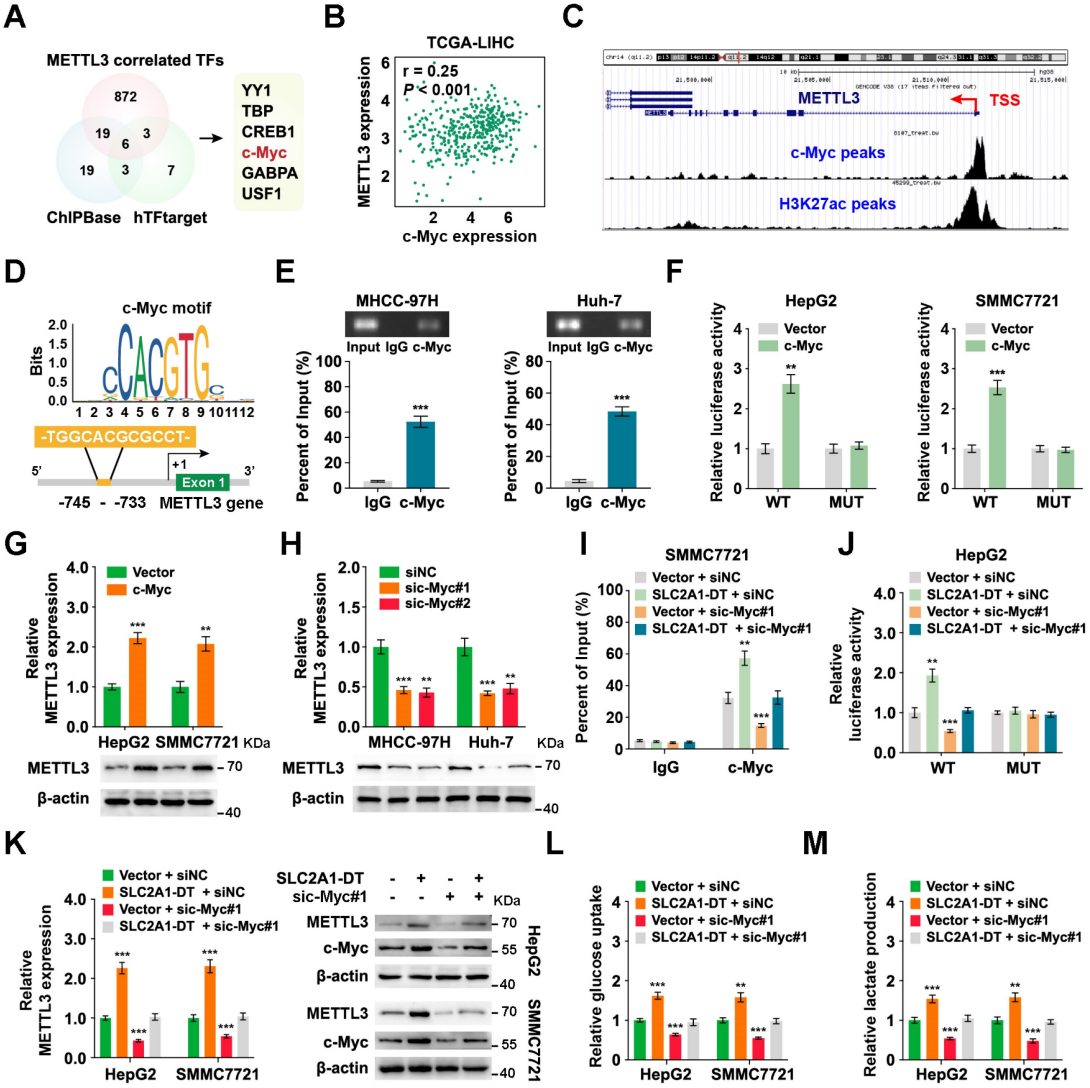
** METTL3 expression is reciprocally regulated by the SLC2A1-DT/c-Myc pathway in HCC. (A)** Venn diagram showing the candidate transcription factors that correlated with METTL3 expression in the CHIPBase (https://rna.sysu.edu.cn/chipbase/) and hTFtarget database (http://bioinfo.life.hust.edu.cn/hTFtarget). **(B)** Correlation analysis between c-Myc and METTL3 from the GEPIA database (http://gepia.cancer-pku.cn/index.html). **(C)** ChIP-seq tracks showing the enrichment of H3K27ac and c-Myc in the METTL3 promoter from the Cistrome Data Browser database (http://cistrome.org/db/#/). **(D)** The c-Myc motif (upper) and the binding site of c-Myc on the METTL3 promoter (lower) were predicted and presented by the JASPAR database (https://jaspar.genereg.net/). **(E)** ChIP assays were employed to identify the relative enrichment of c-Myc (upper) at the METTL3 promoter in HCC cells using anti-c-Myc antibody. Following this, the PCR products were examined via a 2% agarose gel (lower). **(F)** By using luciferase reporter assays and c-Myc binding sites (WT or MUT) located in the METTL3 promoter, the respective activity of c-Myc on the METTL3 promoter in HCC cells was determined. **(G-H)** Western blotting (lower) and qRT-PCR (upper) of HCC cells transfected with the vector, c-Myc, siNC, or sic-Myc, respectively, demonstrate the protein and mRNA levels of METTL3. **(I)** ChIP assays showing the relative enrichment of c-Myc at METTL3 promoter in HCC cells after transfection with the vector or SLC2A1-DT, and co-transfection with siNC or sic-Myc.** (J)** HCC cells co-transfected with siNC or sic-Myc and transfected with the vector or SLC2A1-DT exhibited relative luciferase activities. **(K)** Western blotting (right) and qRT-PCR (left) illustrate the mRNA and protein levels of METTL3 in HCC cells transfected with the indicated agents. **(L-M)** The relative levels of glucose uptake and lactate production were evaluated in HCC cells transfected with as indicated.

To identify the mechanism of upregulation of METTL3 in HCC, we searched the METTL3 correlated transcription factors (TFs) in the TCGA database with the CHIPBase and hTFtarget database (Fig. 7A). Among the six transcription factors in the intersection of the three datasets, the glycolysis related gene, c-Myc 28, was identified as a target for SLC2A1-DT. Therefore, we hypothesized that METTL3 might be reciprocally regulated by c-Myc. Coincidently, data from TCGA database suggested that c-Myc expression was strongly linked with METTL3 level in HCC (Fig. 7B). And the analysis of ChIP-seq tracks from the Cistrome Data Browser database showed H3K27ac and c-Myc enrichments in the METTL3' promoter (Fig. 7C). Meanwhile, the JASPAR database revealed a c-Myc binding sequence on the METTL3 promoter (-745 -TGGCACGCGCCT- -733) (Fig. 7D). Subsequently, the ChIP assay provided evidence that c-Myc could bind to the promoter of METTL3 (Fig. 7E). Additionally, c-Myc overexpression enhanced the promoter activity and METTL3 expression (Fig. 7F-G, Fig. S7A). C-Myc knockdown lowered the promoter activity and METTL3 expression in HCC cells (Fig. 7H, Fig. S7B and C). These results revealed that METTL3 was transcriptionally regulated by c-Myc in HCC cells.

Since our findings indicated that SLC2A1-DT promoted c-Myc expression by enhancing the transactivation of β-catenin, we speculated if SLC2A1-DT could regulate the expression of METTL3. Coincidently, the outcomes of the ChIP assay and luciferase reporter assays displayed that SLC2A1-DT overexpression or inhibition distinctively enhanced or suppressed the enrichment and activation of c-Myc on the promoter of METTL3, which was obviously reversed or rescued by c-Myc knockdown or overexpression (Fig. 7I-J, Fig. S7D and E). Similarly, METTL3 expression, glucose uptake, and lactate production, which were elevated or decreased in SLC2A1-DT overexpressing or depleted cells, were remarkably reversed or rescued by c-Myc silencing or overexpression (Fig. 7K-M, Fig. S7F-H), respectively. Meanwhile, METTL3 inhibition significantly suppressed the protein level of β-catenin and the mRNA/protein level of c-Myc, which was obviously rescued by SLC2A1-DT overexpression (Fig. S7I). Therefore, the above results indicated that METTL3 was reciprocally regulated by the SLC2A1-DT/c-Myc pathway in HCC cells.

### METTL3/SLC2A1-DT/β-catenin/c-Myc axis is necessary for tumorigenesis of HCC

**Figure 8:**
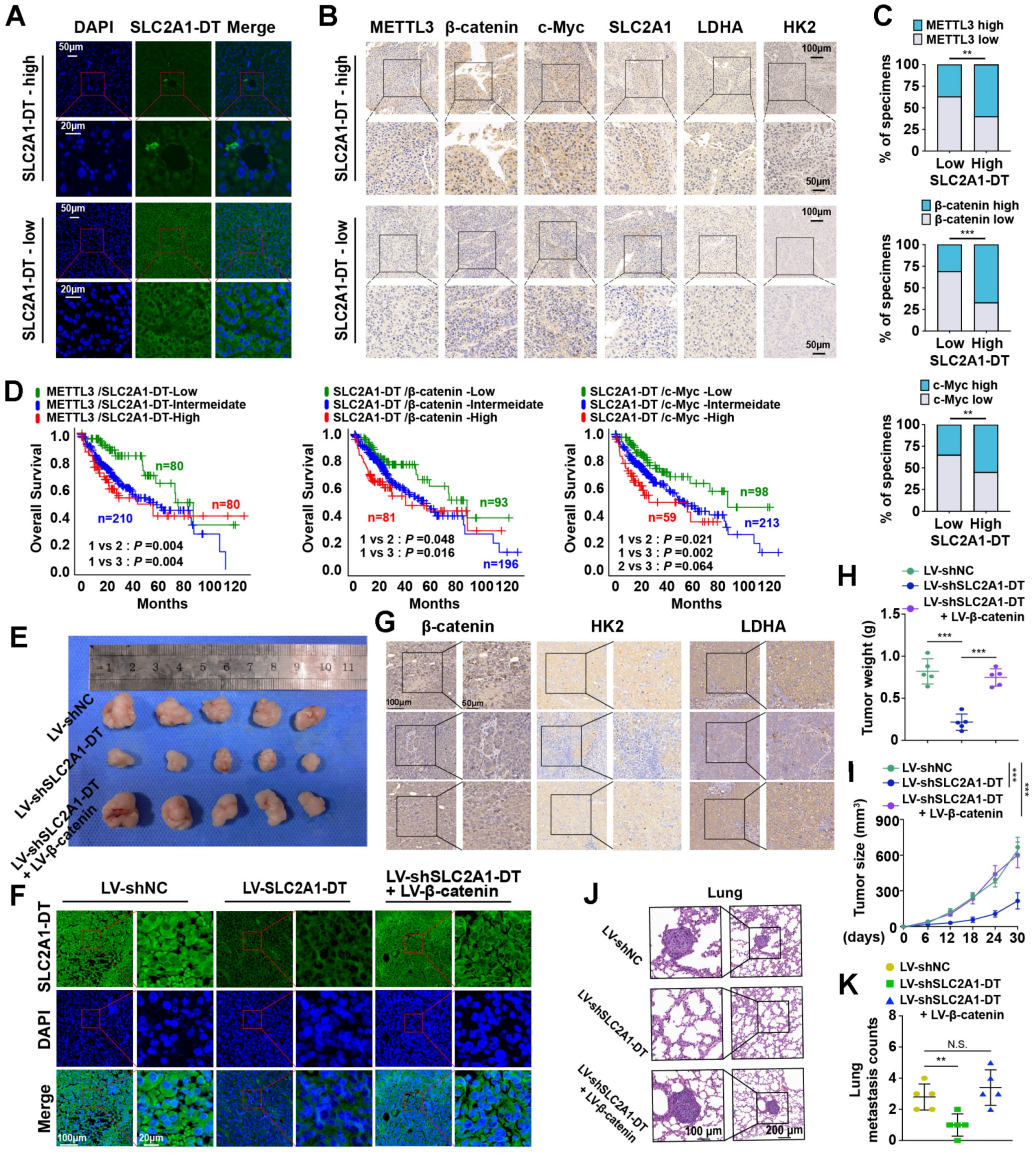
** METTL3/SLC2A1-DT/β-catenin/c-Myc axis is necessary for tumorigenesis of HCC. (A-B)** Representative FISH and IHC images of SLC2A1-DT, METTL3, β-catenin, c-Myc, SLC2A1, LDHA, and HK2 in HCC tissues (n = 32) with low or high levels of SLC2A1-DT. **(C)** Percentages of specimens showing different levels of METTL3, β-catenin and c-Myc in the high or low SLC2A1-DT expression groups (Chi-square test). **(D)** Kaplan-Meier analyses (log-rank test) of the OS curves for HCC patients with different expression levels of SLC2A1-DT, METTL3, β-catenin and c-Myc. **(E)** The images depict xenografts developed via subcutaneous administration of MHCC-97H cells that have been transfected with LV-shNC, LV-shSLC2A1-DT, or co-transfected with LV-β-catenin. **(F-G)** Representative FISH images of SLC2A1-DT, β-catenin, HK2 and LDHA in nude mice subcutaneously injected with MHCC-97H cells transfected with LV-shNC, LV-shSLC2A1-DT or co-transfected with LV-β-catenin. **(H-I)** The volume and weight of tumors formed by subcutaneous injection of MHCC-97H cells transfected with as indicated in nude mice (n = 5/group). **(J-K)** The quantification and representation of lung metastatic colonization in nude mice (n = 5/group) that received MHCC-97H cell injections via the tail vein. NS is not substantial.

Then we assessed the expression of METTL3-SLC2A1-DT-β-catenin axis in HCC tumor samples. The results showed that METTL3, β-catenin, c-Myc, SLC2A1, LDHA and HK2 expression were higher in HCC tumors with high expression of SLC2A1-DT than in HCC tumors with low expression of SLC2A1-DT (Fig. 8A-C, Fig. S8A). Furthermore, the data from TCGA database revealed that HCC patients with higher co-expression of METTL3 and SLC2A1-DT, SLC2A1-DT and β-catenin, SLC2A1-DT and c-Myc, SLC2A1-DT and SLC2A1, SLC2A1-DT and LDHA, as well as SLC2A1-DT and HK2 had worse prognosis (Fig. 8D, Fig. S8B).

To further support the *in vitro* experimental results, MHCC-97H cells were stably transfected with lentivirus containing control, shSLC2A1-DT, or β-catenin. The transfected cells were injected into the dorsal flanks or the tail vein of nude mice to observe the effects of SLC2A1-DT/β-catenin signaling on the propagation and metastasis of HCC cells* in vivo*. The results showed that SLC2A1-DT knockdown obviously inhibited HCC tumor growth, HK2 and LDHA expression, tumor weight, and lung metastasis in nude mice, which was remarkably abolished by β-catenin overexpression (Fig. 8E-K). Collectively, the above results indicate that the SLC2A1-DT-β-catenin axis plays a crucial role in HCC tumorigenesis by enhancing glycolysis.

## Discussion

Abnormal activation of glycolysis, a hallmark of metabolic reprogramming, leads to HCC tumorigenesis *in vitro* 7. Although previous studies have indicated crosstalk between glycolysis and lncRNAs in tumor progression, the potential involvement of lncRNAs in HCC glycolysis remained unexplored. Here, we identified SLC2A1-DT as a glycolysis-associated oncogenic lncRNA in HCC using a combination of bioinformatics analysis and cell biological analyses. The bioinformatics analyses identified that HCC patients with elevated levels of SLC2A1-DT exhibited adverse outcomes and high glycolysis levels. Meanwhile, gain- and loss-of-function experiments showed that SLC2A1-DT was crucial for the propagation, dissemination, and glycolysis of HCC cells. The present study shows evidence that SLC2A1-DT exerts its function by driving YWHAZ-mediated stabilization of β-catenin. β-catenin accumulation contributes to the transcriptional activation of glycolysis-related genes (c-Myc, SLC2A1, LDHA, and HK2), thereby resulting in enhanced glycolysis, proliferation and metastasis in HCC. In addition, METTL3/YTHDF1, the m6A writer and reader, was found to be responsible for the upregulation of SLC2A1-DT in a m6A-dependent manner. Furthermore, the SLC2A1-DT mediated upregulation of c-Myc, a transcriptional factor, enhanced the expression of METTL3. As a result, the METTL3/ SLC2A1-DT/β-catenin/ c-Myc axis forms a feedback loop, promoting glycolysis, proliferation and metastasis of HCC.

Recent studies have reported SLC2A1-DT as a novel lncRNA and that it is overexpressed in esophageal squamous cell carcinoma (ESCC) and lung adenocarcinoma (LUAD) patients 29, 30. The above studies have revealed that SLC2A1-AS1 exerts its effects on enhancing aerobic glycolysis and oncogenesis in ESCC by absorbing miR-378a-3p, thereby triggering the accumulation of Glut1 30. Here, using bioinformatics analyses, we show that SLC2A1-DT is overexpressed in HCC. The HCC patients with enhanced levels of SLC2A1-DT exhibited adverse prognosis. Depletion of SLC2A1-DT suppressed the propagation, invasion, migration and glycolysis of HCC cells. Upregulation of SLC2A1-DT had the opposite effects. Based on our findings, elevated SLC2A1-DT expression substantially influenced glycolysis and tumorigenesis in HCC.

The current study indicated that SLC2A1-DT was mainly localized in the cytoplasm. Cytoplasmic lncRNAs are known to be involved in regulating protein stability and modification 31. Our results indicated that SLC2A1-DT is bound to YWHAZ. YWHAZ is known to be an oncogene in a variety of cancers, including HCC 32. Meanwhile, high expression of YWHAZ enhanced cell proliferation, metastasis, EMT, chemoresistance and decreased apoptosis in HCC. However, the relationship between YWHAZ and glycolysis has not yet been explored in depth. β-catenin, a multifunctional protein with critical roles in physiological homeostasis, is overexpressed in cancer and promotes tumor progression by multiple pathways 33. The upregulated β-catenin translocates into the nucleus and activates aerobic glycolysis by enhancing the expression of c-Myc and glycolytic enzymes, including lactate dehydrogenase A (LDH-A), HK2 and glucose transporter (SLC2A1) 23, 34. Recently, studies revealed that YWHAZ overexpression enhances the β-catenin-mediated transcription by upregulating β-catenin levels in the cytosol and nucleus 21. The above study demonstrated that YWHAZ bound to β-catenin, which resulted in the disassociation of β-catenin from the E3 ubiquitin ligase β-TrCP and enhanced β-catenin stability. We observed that SLC2A1-DT positively regulated the protein stability of β-catenin with no effect on its mRNA expression level. Furthermore, the Co-IP assay indicated that SLC2A1-DT enhanced the connection between YWHAZ and β-catenin, resulting in the disassociation of β-catenin from β-TrCP and an increase in β-catenin stability. Additionally, the upregulation of β-catenin induced its translocation to the nucleus, activating glycolysis and tumor progression by inducing the expression of c-Myc and glycolytic enzymes. The above data consistently indicated that SLC2A1-DT exerted its function by driving YWHAZ-mediated stabilization of β-catenin, resulting in enhanced glycolysis, proliferation, and metastasis of HCC.

We noted that m6A methylation has been elevated in SLC2A1-DT cells of HCC via m6A RIP experiments. Further, knockdown and overexpression of METTL3 decreased and increased the m6A methylation and RNA stability of SLC2A1-DT, respectively. Moreover, YTHDF1, the m6A writer, also positively regulated the expression and stability of SLC2A1-DT in a m6A-dependent manner. Therefore, the increased levels of SLC2A1-DT in HCC may be related to the m6A modification.

Being an essential methyltransferase for m6A modification, METTL3 regulates the RNA level in a m6A-dependent manner and drives drug resistance and tumor progression in HCC 35. However, the mechanism underlying the dysregulation of METTL3 expression in HCC has not yet been fully investigated. Previously, a study revealed that cigarette smoke condensate promoted METTL3 overexpression by inducing hypomethylation of METTL3 promoter and recruitment of the transcription factor NFIC in pancreatic cancer 36. Moreover, it was shown in gastric cancer that the upregulation of METTL3 was induced by P300-mediated H3K27 acetylation of METTL3 promoter 37. Meanwhile, another study indicated that the microRNA miR-242-2 enhanced METTL3 transcription 38. Here, we identified that c-Myc, which was induced by SLC2A1-DT-mediated upregulation of β-catenin, elevated the expression of METTL3 by activating the METTL3 transcription, forming a feedback loop. Thus, the METTL3/ SLC2A1-DT /β-catenin axis is crucial for glycolysis and HCC tumorigenesis.

In summary, these findings highlight that the m6A modification mediated by SLC2A1-DT is closely associated with glycolysis and HCC progression. Mechanistically, the lncRNA SLC2A1-DT is upregulated by m6A modification and activates glycolysis, and promotes HCC progression by stabilizing β-catenin. The METTL3/SLC2A1-DT/β-catenin axis is crucial for glycolysis and HCC tumorigenesis, and targeting this pathway may act as a possible treatment option for HCC patients.

## Supplementary Material

Supplementary figures and tables.

## Abbreviations

LncRNAs: Long noncoding RNAs
HCC: Hepatocellular carcinoma
METTL3: methyltransferase 3
LDH-A: Lactate dehydrogenase A
HK2: Hexokinase 2
SLC2A1: Glucose transporter
18F-FDG: 18F-fludeoxyglucose
PET: positron emission tomography
M6A: N6-methyladenosine
RIP: RNA binding protein immunoprecipitation
MeRIP: RNA immunoprecipitation
qRT-PCR: quantitative real-time PCR
RNA-FISH: RNA fluorescence *in situ* hybridization
DAPI: 4,6-diamidino-2-phenylindole
LSCM: Laser Scanning Confocal Microscope
Co-IP: Co-immunoprecipitation
IHC: Immunohistochemistry
ChIP: Chromatin immunoprecipitation
GSEA: Gene set enrichment analysis
GO: Gene ontology
KEGG: Kyoto Encyclopedia of Genes and Genomes
OS: overall survival
DFI: disease-free interval
DSS: disease-specific survival
PFI: Progression-free interval
HR: hazard rate
SO: Sodium oxamate
YWHAZ: tyrosine 3-monooxygenase/tryptophan 5-monooxygenase activation protein zeta
CHX: cycloheximide
ROC: receiver operating characteristic
TFs: transcription factors
ESCC: esophageal squamous cell carcinoma
LUAD: lung adenocarcinoma
