# Supplementary material for: The m6A modification-mediated positive feedback between glycolytic lncRNA SLC2A1-DT and c-Myc promotes tumorigenesis of hepatocellular carcinoma
Source: Int J Biol Sci. 2024 Feb 25;20(5):1744–62. doi: 10.7150/ijbs.86658 (PMC10929188; doi:10.7150/ijbs.86658)
Supplement: Supplementary file 1 — Supplementary figures and tables. [file ijbsv20p1744s1.pdf]

Figure S1

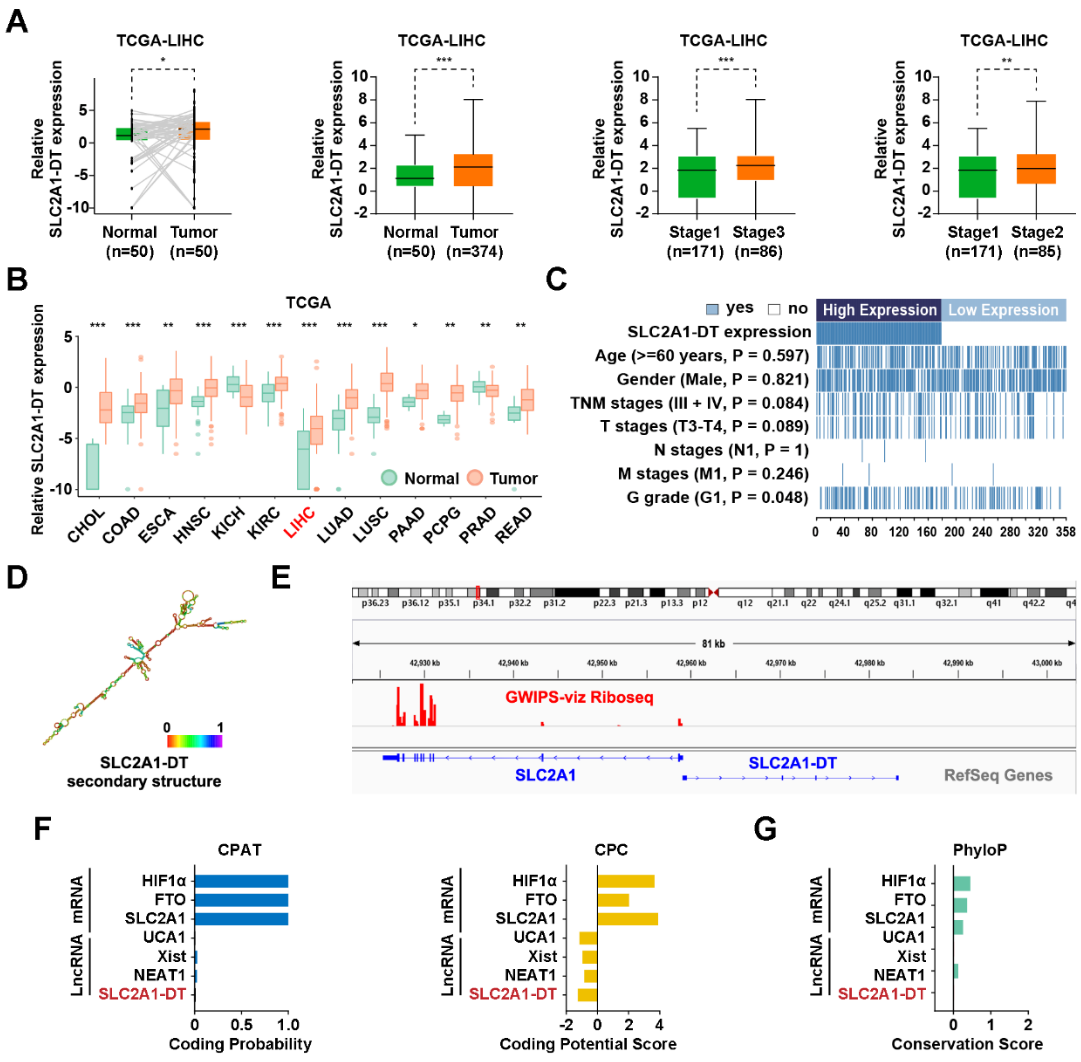

Figure S1. SLC2A1-DT was upregulated in HCC.

(**A**) Boxplot showing the differential expression of SLC2A1-DT in the normal and tumor tissues with different clinical stages.

(**B**) The overall expression of SLC2A1-DT in multiple human tumors from TCGA database.

(**C**) The heatmap showing the association of different clinical characters with SLC2A1-DT high or low expression. $\chi2$ test was used in the statistical analyses.

(**D**) The predicted secondary structure of SLC2A1-DT.

(**E**) Ribosome profiling data (https://gwips.ucc.ie/index.html) presented by the Integrative genomics viewer (IGV) showing the low protein-coding potential of SLC2A1-DT.

(**F**) The low coding capacity of SLC2A1-DT was predicted by CPAT (http://lilab.research.bcm.edu/cpat/index.php) and CPC (http://cpc2.cbi.pku.edu.cn/), respectively. The coding capacity of known protein-coding mRNAs (HIF-1α, FTO, SLC2A1) and lncRNAs (UCA1, Xist, NEAT1) were used as control.

(**G**) The mean conservation score of SLC2A1-DT was analyzed by PhyloP across 100 vertebrates. The protein-coding mRNAs (HIF-1α, FTO, SLC2A1) and lncRNAs (UCA1, NEAT1) were used as controls. All data were presented as means ± SD.

Figure S2

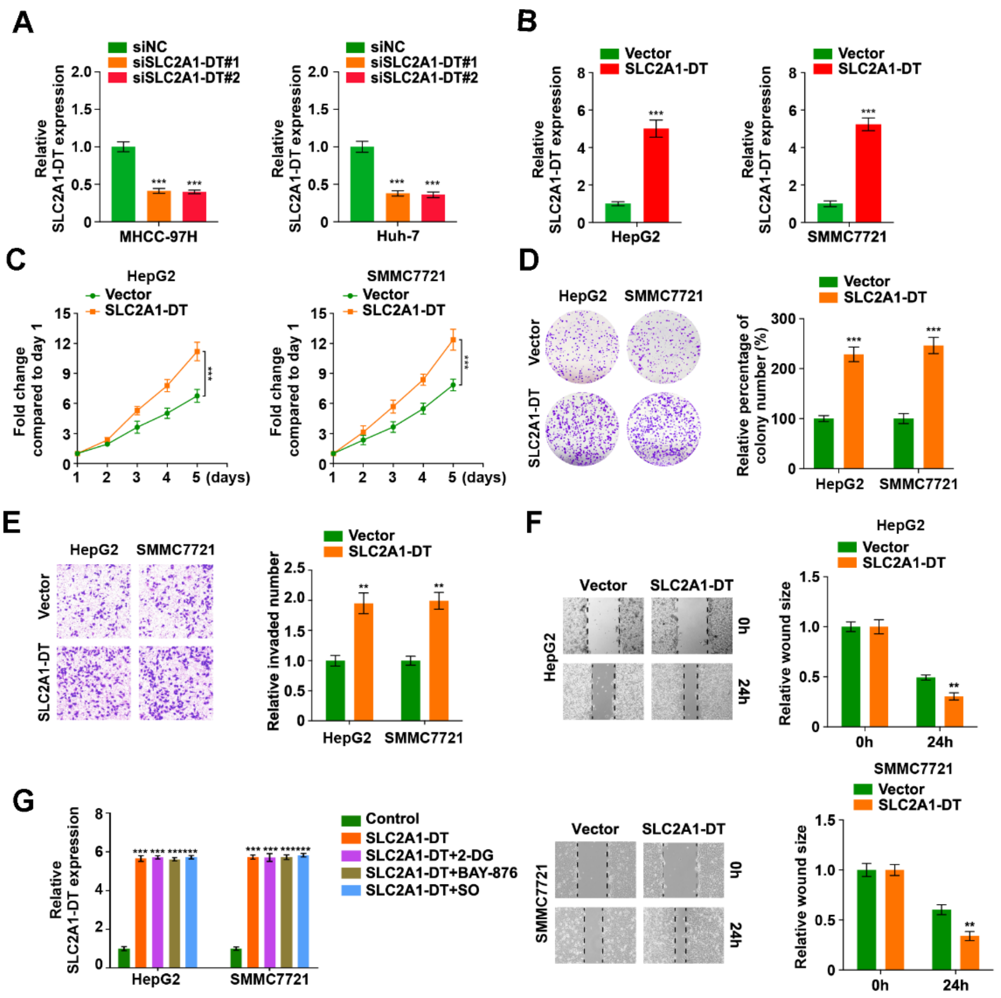

Figure S2. SLC2A1-DT promotes proliferation, metastasis, invasion by enhancing glycolysis of HCC cells.

(**A**) qRT-PCR analysis of SLC2A1-DT expression level was performed in MHCC-97H and Huh-7 cells after their transfection with specific siRNA (siSLC2A1-DT) and negative control (siNC).

(**B**) SLC2A1-DT level was evaluated by qRT-PCR in HepG2 and SMMC7721 cells after transfected with pcDNA-SLC2A1-DT plasmid (SLC2A1-DT) and its negative control (Vector), respectively.

(**C**) Cell growth curves were measured by MTT colorimetric assay in HepG2 and SMMC7721 cells after transfected with SLC2A1-DT or control vector, respectively.

(**D**) Colony formation assays (left) and relative quantification (right) showing the growth of cells transfected with SLC2A1-DT vector.

(**E**) Transwell assay (up) and relative quantification (down) were applied in HepG2 and SMMC7721 cells after transfected with SLC2A1-DT plasmids.

(**F**) Wound-healing assay was performed in HepG2 and SMMC7721 cells after transfected with SLC2A1-DT. Representative images (left) and relative wound sizes (right) were shown.

(**G**) SLC2A1-DT level was evaluated by qRT-PCR in overexpression HepG2 and SMMC7721 cells with SLC2A1-DT augmentation and 2-DG, BAY-876 or Sodium oxamate treatment. All data were presented as means ± SD of at least three independent experiments.

Figure S3

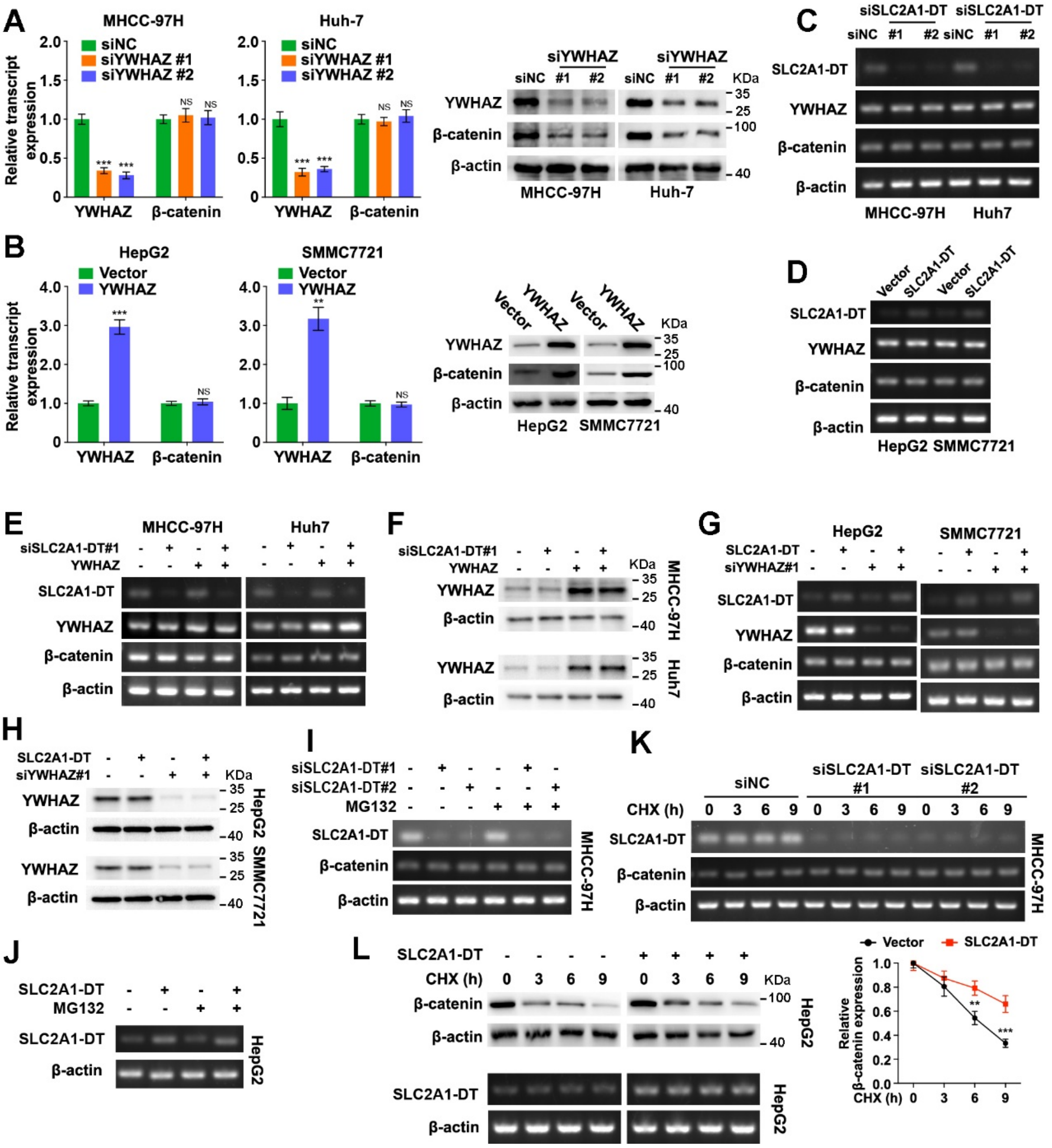
A
MHCC-97H
Huh-7
siNC
siYWHAZ #1
siYWHAZ #2
Relative transcript expression
YWHAZ
β-catenin
MHCC-97H
Huh-7
B
HepG2
SMMC7721
Vector
YWHAZ
C
siSLC2A1-DT
SLC2A1-DT
Huh7
D
E
F
G
siSLC2A1-DT#1
siYWHAZ#1
H
I
MG132
J
K
CHX (h)
L
β-actin
KDa
β-catenin expression

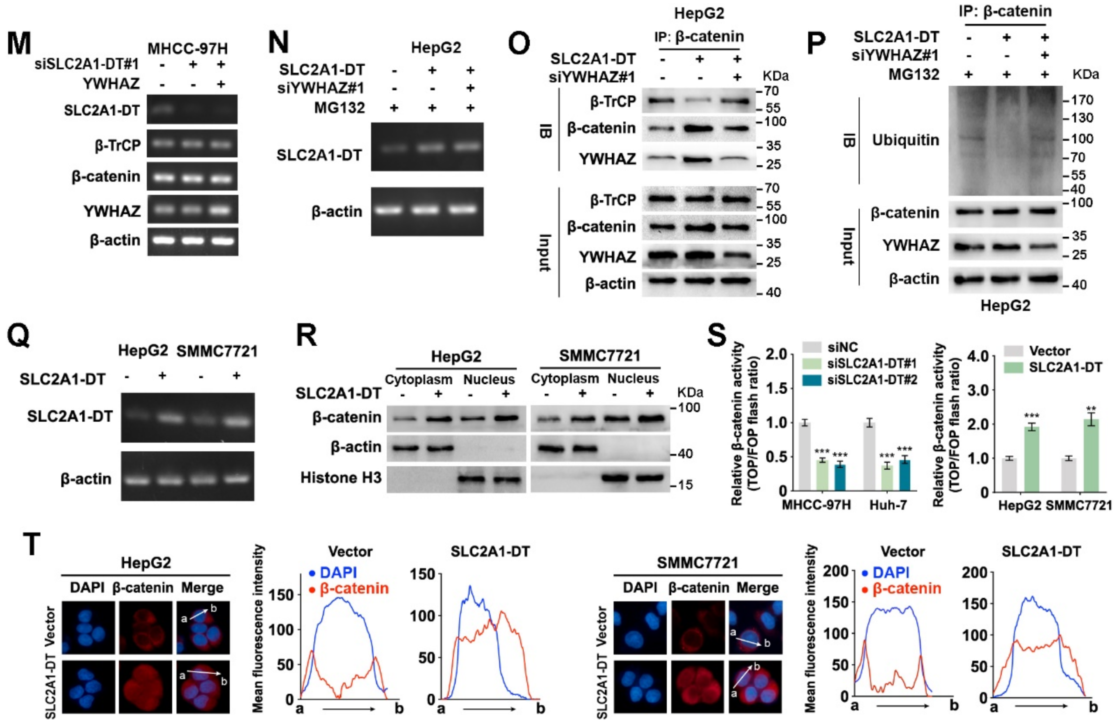

Figure S3. SLC2A1-DT/YWHAZ signaling facilitates the stability of β-catenin in HCC cells.

(**A-B**) The RNA and protein level of YWHAZ and β-catenin were detected by qRT-PCR (left) and western blot (right) in HCC cells with or without YWHAZ knockdown or overexpression.

(**C-E**) Semi-quantitative RT-PCR assay showing SLC2A1-DT expression and the mRNA levels of YWHAZ and β-catenin in MHCC-97H and Huh-7 cells transfected with indicated siRNA or plasmids.

(**F**) Western blot assay showing the protein level of YWHAZ in MHCC-97H and Huh-7 cells transfected with siNC or siSLC2A1-DT and co-transfected with vector or YWHAZ plasmids.

(**G**) Semi-quantitative RT-PCR assay showing SLC2A1-DT expression and the mRNA levels of YWHAZ and β-catenin in HepG2 and SMMC7721 cells transfected with

vector or SLC2A1-DT plasmids and co-transfected with siNC or siYWHAZ.

(**H**) Western blot assay showing the protein level of YWHAZ in HepG2 and SMMC7721 cells transfected with vector or SLC2A1-DT plasmids and co-transfected with siNC or siYWHAZ.

(I-J) Semi-quantitative RT-PCR assay showing SLC2A1-DT expression and the mRNA levels of β-catenin in MHCC-97H cells transfected with siSLC2A1-DT and HepG2 cells transfected with SLC2A1-DT plasmids for 48h followed by harvesting the cells for western blotting for β-catenin after treatment with or without MG132 (10 μmol/L).

(K) Semi-quantitative RT-PCR assay showing SLC2A1-DT expression and the mRNA levels of β-catenin in MHCC-97H cells transfected with siSLC2A1-DT for 48h followed by treatment with Cycloheximide (CHX, 12.5 g/mL) at indicated time points.

(L) (Up) HepG2 cells were transfected with SLC2A1-DT plasmid and vector for 48h followed by treatment with Cycloheximide (CHX, 12.5 g/mL) and then collected at different time points for western blotting (left panel). The relative intensity (right panel) of protein bands was measured using the ImageJ software. (Down) Semi-quantitative RT-PCR assay showing SLC2A1-DT expression in HepG2 cells transfected with SLC2A1-DT plasmid and vector for 48h followed by treatment with Cycloheximide (CHX, 12.5 g/mL) at indicated time points.

(M) Semi-quantitative RT-PCR assay showing SLC2A1-DT expression and the mRNA levels of β-TrCP, β-catenin and YWHAZ in MHCC-97H cells transfected with siSLC2A1-DT and co-trasfected with YWHAZ plasmid.

(N) Semi-quantitative RT-PCR assay showing SLC2A1-DT expression in HepG2 cells

transfected with SLC2A1-DT plasmid and co-trasfected with siYWHAZ followed by treatment with MG132 (10 μmol/L).

(**O**) After transfected with SLC2A1-DT plasmids or co-transfected with siYWHAZ, HepG2 cell lysates were immunoprecipitated with anti-β-TrCP, anti-β-catenin, and anti-YWHAZ antibodies.

(**P**) HepG2 cells were transfected with SLC2A1-DT plasmids or co-transfected with siYWHAZ. After 48 h, cell lysates were collected for western blot assays after being treated with MG132. And the protein lysates were immunoprecipitated with anti-β-catenin, anti-YWHAZ, and anti-ubiquitin antibodies.

(**Q**) Semi-quantitative RT-PCR assay showing SLC2A1-DT expression in HepG2 and SMMC7721 cells transfected with SLC2A1-DT plasmid.

(**R**) Western blot analysis of β-catenin in the cytoplasmic and nucleus portions of SLC2A1-DT overexpressed cells.

(**S**) Immunofluorescence images (left panel) indicating the localization of β-catenin in HepG2 and SMMC7721 cells transfected with vector or SLC2A1-DT. The mean fluorescence intensities between points a and b were measured by ImageJ software (right panel).

(**T**) Dual-luciferase assays indicating the relative activity of β-catenin in HCC cells transfected with siNC, siSLC2A1-DT, vector or SLC2A1-DT plasmids. All data were presented as means ± SD of at least three independent experiments.

Figure S4

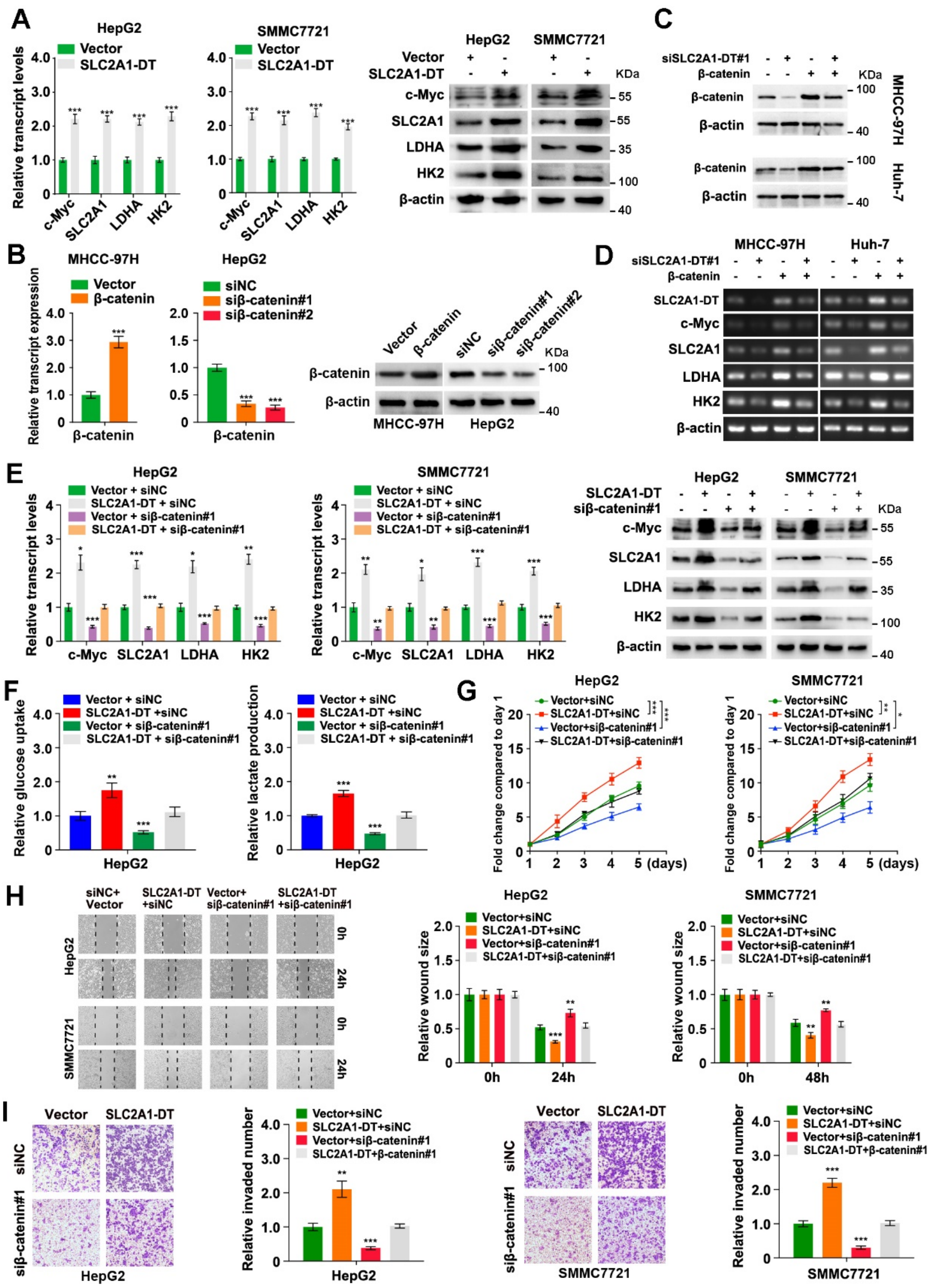

Figure S4. SLC2A1-DT/ β-catenin enhances glycolysis by regulating glycolytic genes.

(**A**) The mRNA (left panel) and protein (right panel) levels of c-Myc and downstream

genes (SLC2A1, LDHA and HK2) in HepG2 and SMMC7721 cells transfected with vector or SLC2A1-DT plasmids.

(**B**) The transfection efficiencies of β-catenin in HepG2 and SMMC7721 cells.

(**C**) The protein levels of β-catenin in HCC cells transfected with vector or siSLC2A1-DT and co-transfected with β-catenin plasmids.

(**D**) The SLC2A1-DT expression and mRNA levels of c-Myc and downstream genes, including SLC2A1, LDHA and HK2 in HCC cells transfected with siSLC2A1-DT and co-transfected with β-catenin plasmids.

(**E**) After transfected with vector, SLC2A1-DT, or co-transfected with siNC or siβ-catenin, the mRNA (left panel) and protein (right panel) levels of c-Myc and downstream genes (SLC2A1, LDHA and HK2) in HCC cells were detected.

(**F**) The glucose uptake (left panel) and the lactate production levels (right panel) were detected in HepG2 cells transfected with vector or SLC2A1-DT plasmids, and those co-transfected with siNC or siβ-catenin.

(**G-I**) The relative proliferation, migration and invation abilities were detected by MTT, wound healing, and transwell assays in HCC cells, respectively, transfected with vector or SLC2A1-DT plasmids, and those co-transfected with siNC or siβ-catenin. All data were presented as means ± SD of at least three independent experiments.

Figure S5

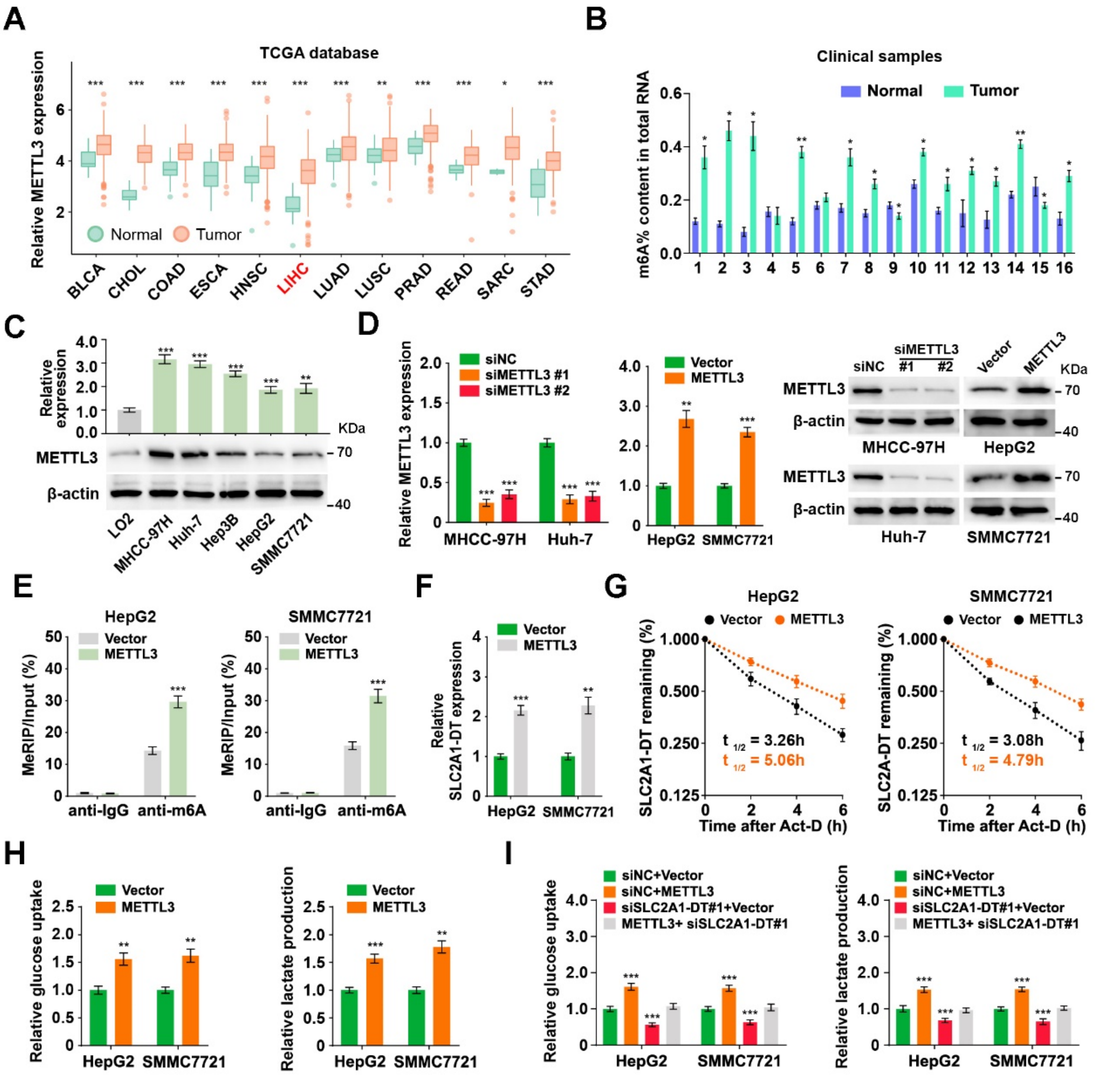

Figure S5. Associated figures of figure 6.

(**A**) The Pan-cancer analysis of METTL3 expression in TCGA database.

(**B**) The m6A content of 16 pairs of tumor and normal tissues.

(**C**) The mRNA and protein levels of METTL3 in five HCC cell lines (MHCC-97H, Huh-7, Hep3B, HepG2, SMMC-7721) and normal hepatocyte cells (LO2).

(**D**) The qRT-PCR (left panel) and western blot (right panel) showing the expression of METTL3 in HCC cells transfected with siNC, siMETTL3, empty vector, or METTL3 plasmids.

(**E**) The MeRIP-qPCR showing the effects of METTL3 overexpression on the enrichment of m6A with SLC2A1-DT.

(**F**) The qRT-PCR showing the SLC2A1-DT levels in HCC cells transfected with vector or METTL3 plasmids.

(**G**) The RNA stabilities of SLC2A1-DT in HCC cells transfected with vector or METTL3 plasmids.

(**H**) The relative glucose uptakes (left panel) and lactic acid levels in media (right panel) were measured in HepG2 and SMMC7721 cells transfected with vector or METTL3 plasmids.

(**I**) After transfected with vector or METTL3 plasmids, and those co-transfected with siNC or siSLC2A1-DT, the relative glucose uptakes (left panel) and lactate production (right panel) were evaluated in HCC cells. All data were presented as means ± SD of at least three independent experiments.

Figure S6

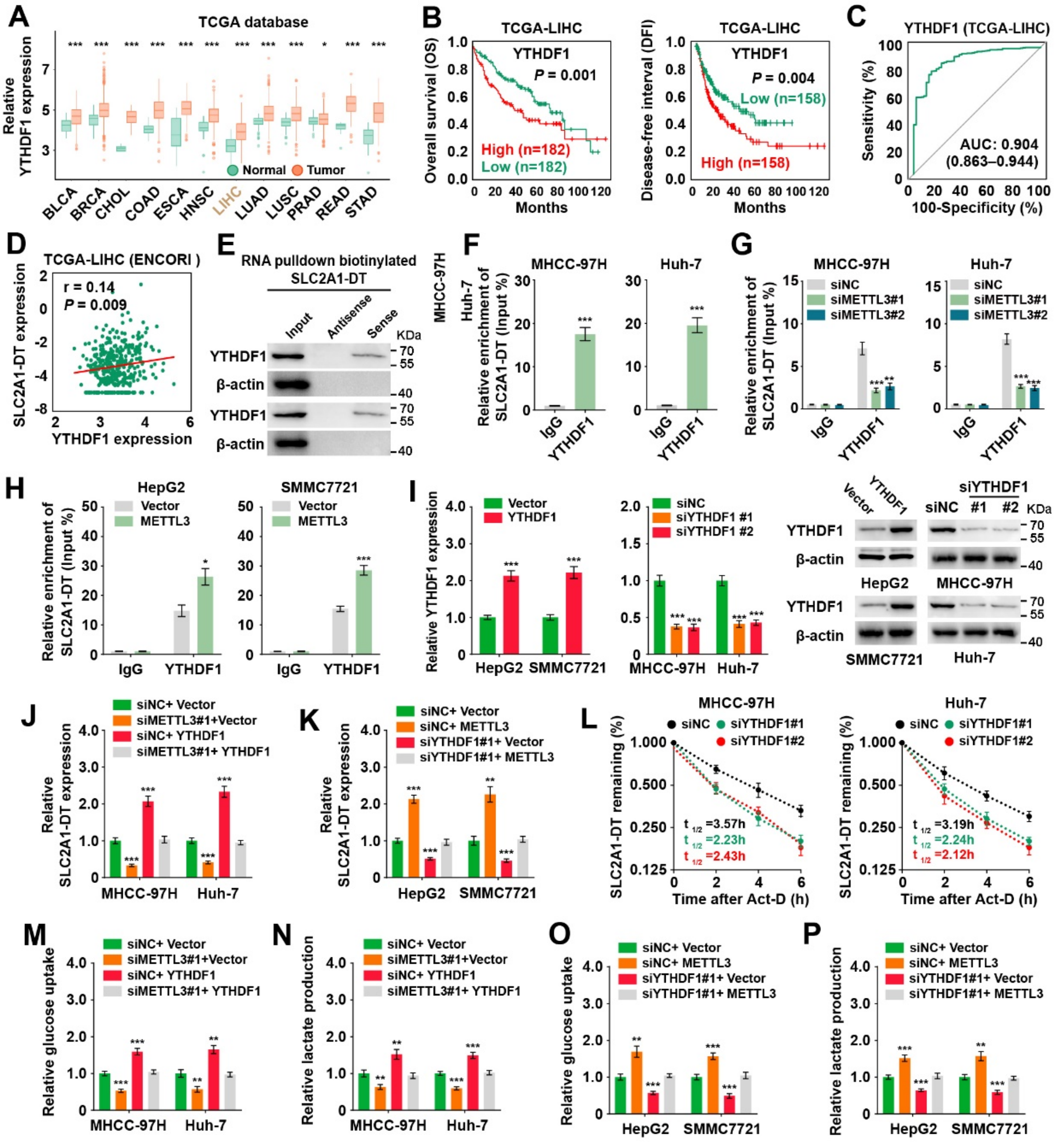

Figure S6. YTHDF1 is required for m6A mediated stabilization of SLC2A1-DT

(**A**) The overall survival (OS) and disease free survival (DFI) were analyzed and compared between patients with high and low levels of YTHDF1 expression in the TCGA-LIHC dataset.

(**B**) Pan-cancer analysis of YTHDF1 levels in the TCGA database.

(**C**) ROC curve of YTHDF1 (AUC = 0.904, 95% CI: 0.863 to 0.944) in the TCGA-LIHC dataset.

(**D**) Correlation between YTHDF1 and METTL3 expression in HCC patients from the ENCORI database.

(**E**) YTHDF1 was pulled down by biotin-labeled sense RNA of SLC2A1-DT but not by SLC2A1-DT antisense RNA in HCC cells.

(**F**) RIP assays showed the SLC2A1-DT enrichment on YTHDF1 using the anti-YTHDF1 antibody in HCC cells.

(**G**, **H**) RIP assays were applied in HCC cells transfected with siNC, siMETTL3, vector or METTL3 using the anti-YTHDF1 antibody, followed by qRT-PCR analysis of SLC2A1-DT enrichment.

(**I**) The mRNA and protein level of YTHDF1 in YTHDF1 knockdown or overexpression HCC cells.

(**J, K**) qRT-PCR analysis of SLC2A1-DT levels in HCC cells transfected as indicated.

(**L**) HCC cells were transfected with siNC or siYTHDF1 for 48h, and further treated with actinomycin D (Act-D) for the indicated duration. The relative levels of SLC2A1-DT expression was measured by qRT-PCR.

(**M, N**) The relative glucose uptake and lactic acid production levels in media were evaluated in HCC cells transfected with siNC or siMETTL3, and those co-transfected with vector or YTHDF1.

(**O, P**) The relative glucose uptakes and lactic acid levels in media were evaluated in HCC cells transfected with siNC or siYTHDF1, and those co-transfected with vector or METTL3. All data were presented as means ± SD of at least three independent experiments.

Figure S7

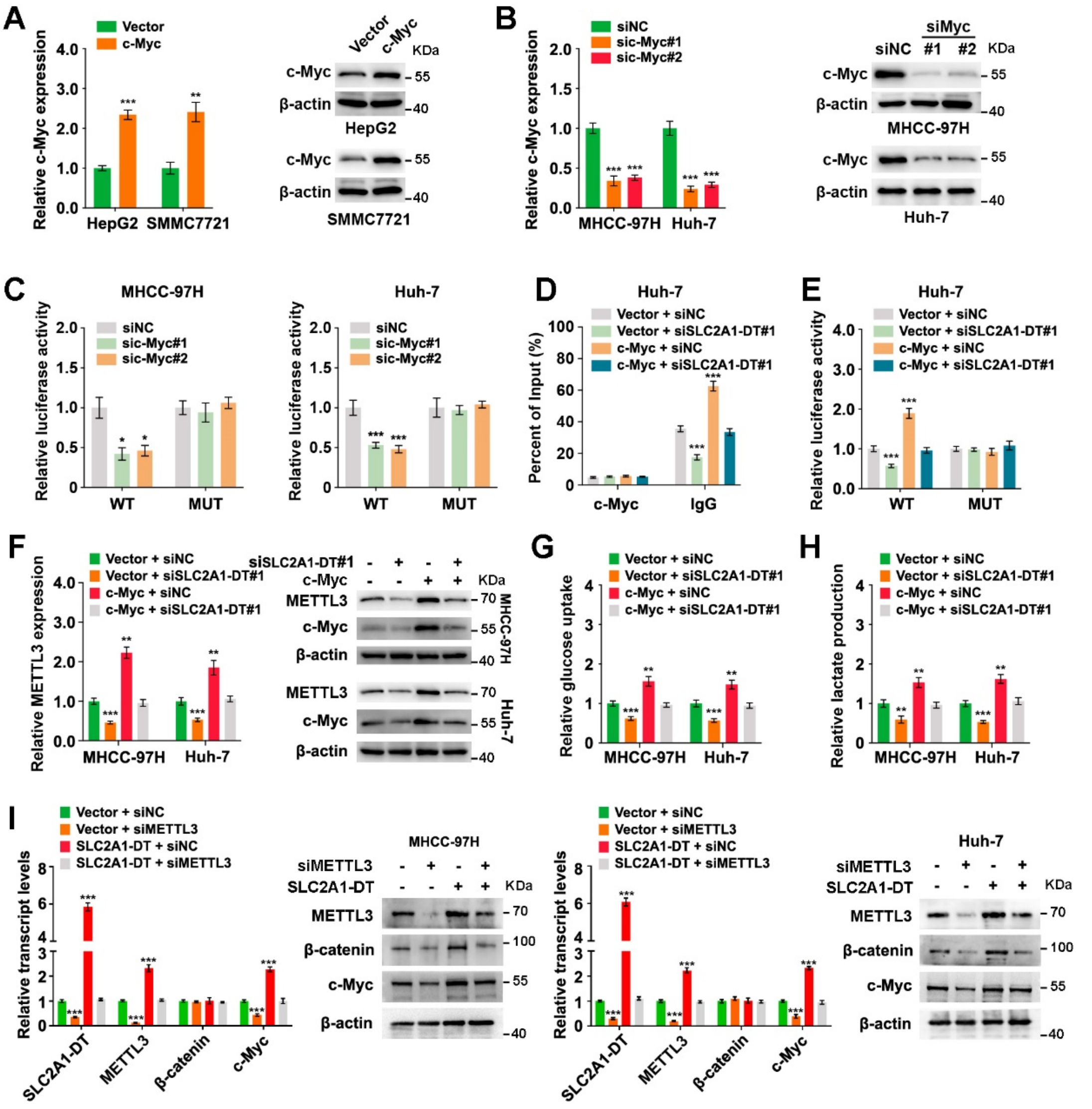

Figure S7. METTL3 expression is reciprocally regulated by SLC2A1-DT/c-Myc pathway in HCC.

(**A-B**) After transfected with vector, c-Myc, siNC or sic-Myc, the mRNA (left panel) and protein (right panel) levels of c-Myc were evaluated in HCC cells.

(**C**) After knockdown of c-Myc, the transcriptional activity of c-Myc on METTL3 promoter was determined by luciferase assays using wild type (WT) or mutant-type (MUT) of c-Myc binding sites.

(**D**) Whole cell lysates from HCC cells transfected with as indicated were subjected to

ChIP assays. ChIP products were analyzed by qRT-PCR using specific primers directed to the fragments of the METTL3 promoter.

(**E**) HCC cells transfected with as indicated. The transcriptional activity of c-Myc on METTL3 promoter was determined via luciferase assays using wild type (WT) or mutant-type (MUT) of c-Myc binding sites.

(**F**) The mRNA and protein lysates from HCC cells transfected with as indicated were subjected to qRT-PCR (left panel) and western blot (right panel).

(**G-H**) After transfected with vector or c-Myc, and those co-transfected with siNC or siSLC2A1-DT, the relative glucose uptakes and lactate production were evaluated in HCC cells. All data were presented as means ± SD of at least three independent experiments.

(**I**) The mRNA and protein lysates from HCC cells transfected with siNC or siMETTL3 and co-transfected with vector or SLC2A1-DT were subjected to qRT-PCR (left panel) and western blot (right panel).

Figure S8

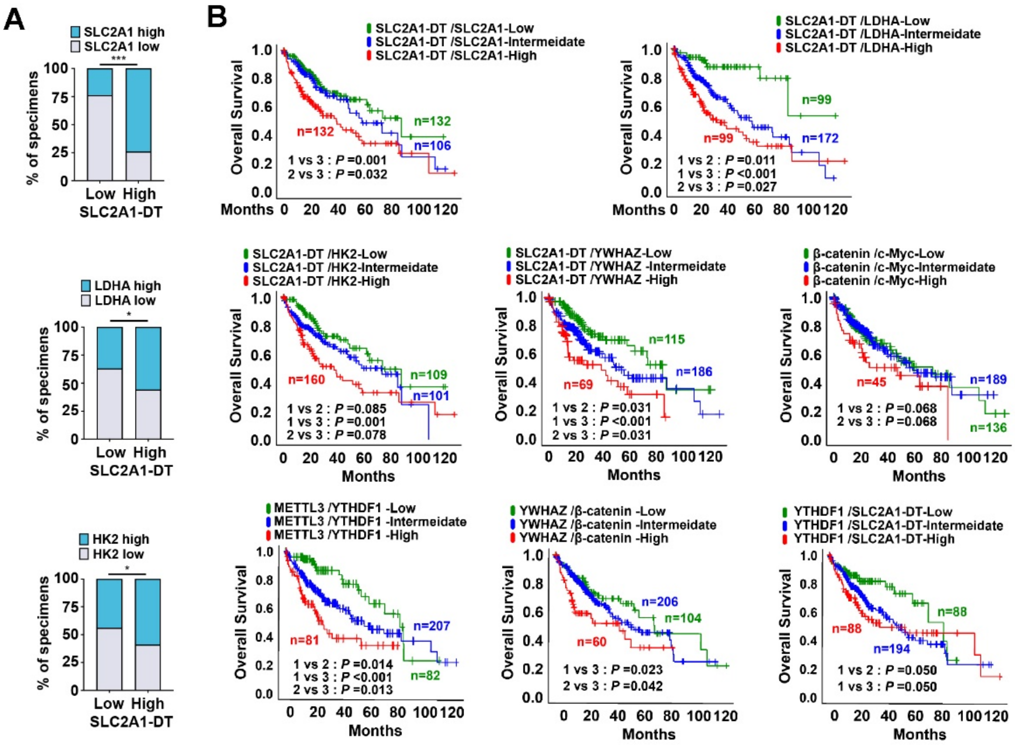

Figure S8. Related figure for Figure 8.

(A) Percentages of specimens showing different levels of SLC2A1, LDHA and HK2 in the high or low SLC2A1-DT expression groups (Chi-square test).

(B) Kaplan-Meier analyses (log-rank test) of the overall survival curves for HCC patients with different expression levels of SLC2A1-DT, SLC2A1, LDHA, HK2, YWHAZ, β-catenin, METTL3, YTHDF1 and c-Myc.

Table S1. The sequences of PCR primers.

Primer	Sequence
SLC2A1-DT	Forward: 5′- CTCCTTCTGTAGGCTTTGT -3′
	Reverse: 5′- ATTATTCAGGTTGGGTGC -3′
U6	Forward: 5′- CTCGCTTCGGCAGCACA -3′
	Reverse: 5′- AACGCTTCACGAATTTGCGT -3′
β-actin	Forward: 5′- AAGGATTCCTATGTGGGCGAC -3′
	Reverse: 5′- GCTTGTTACTATATGCTTTTTAAAT -3′
YWHAZ	Forward: 5′- CCTGCATGAAGTCTGTAACTGAG -3′
	Reverse: 5′- GACCTACGGGCTCCTACAACA -3
β-catenin	Forward: 5’- CAACTAAACAGGAAGGGATG -3’
	Reverse: 5’- CACAGGTGACCACATTTATATC -3’
c-Myc	Forward: 5’- GGCTCCTGGCAAAAGGTCA -3’
	Reverse: 5’- CTGCGTAGTTGTGCTGATGT -3’
LDHA	Forward: 5’- AGGCTATTCTTGGGCAACCC -3’
	Reverse: 5’- TGAGTAGACATCCACCAAGGTT -3’
SLC2A1	Forward: 5′- ATTGGCTCCGGTATCGTCAAC-3′
	Reverse: 5′- GCTCAGATAGGACATCCAGGGTA -3′
LDHA	Forward: 5′- AGGCTATTCTTGGGCAACCC-3′
	Reverse: 5′- TGAGTAGACATCCACCAAGGTT-3′
METTL3	Forward: 5′- TTGTCTCCAACCTTCCGTAGT -3′
	Reverse: 5′- CCAGATCAGAGAGGTGGTGTAG -3′

YTHDF1	Forward: 5′- ATACCTCACCACCTACGGACA -3′
	Reverse: 5′- GTGCTGATAGATGTTGTTCCCC-3′
METTL3-CHIP	Forward: 5′- GCCTGGGCAGTAAGAATG -3′
	Reverse: 5′- ACAAGGTTGGTGGTGGTG -3′
SLC2A1-DT-probe	Forward: 5′- TGTAGAAAGACCAAGTGA -3′
	Reverse: 5′- TAATACGACTCACTATAGGGCTGTGAGAAGTAGAGGAA -3′
SLC2A1-DT-transcription in vitro	Forward: 5′- TAATACGACTCACTATAGGGTGTAAGGCAAGCT -3′
	Reverse: 5′- GCCATTTTTTACCCTTTAGTTCAGATGTATTTC -3′
P1	Forward: 5′- TAATACGACTCACTATAGGGACACAGCAGAAA-3′
	Reverse: 5′- CGTTTTGCATTTAGACTTACC -3′
P2	Forward: 5′- TAATACGACTCACTATAGGGACATTCCCGTTT-3′
	Reverse: 5′- TGCGTGGTTCCGTGCTG -3′
P3	Forward: 5′- TAATACGACTCACTATAGGGGCCTCGCTCAGG-3′
	Reverse: 5′- TGGCTCACAAACGGGAATG -3′

Table S2. The sequences for gene knockdown.

SiRNA Targets	Sequences
SLC2A1-DT-siRNA#1 sense	5′- GCAAUACCAGAGUCUGCAUTT -3′
SLC2A1-DT -siRNA#2 sense	5′- CCAUCUCAAACCUGGGCUUTT -3′
YWHAZ-siRNA#1 sense	5′- GCCUGCAUGAAGUCUGUAATT -3′
YWHAZ-siRNA#2 sense	5′- CGUCUCAAGUAUUGAACAATT -3′
β-catenin-siRNA#1 sense	5′- GCUGCUUUAUUCUCCCAUUTT -3′
β-catenin-siRNA#2 sense	5′- GGACACAGCAGCAAUUUGUTT -3′
METTL3-siRNA#1 sense	5′- GCUCAACAUACCCGUACUATT -3′
METTL3-siRNA#2 sense	5′- GGUUGGUGUCAAAGGAAAUTT -3′
c-Myc-siRNA#1 sense	5′- AAAAAUCCAGCGUCUAAGCAGTT-3′
c-Myc-siRNA#2 sense	5′- AACAACAUCGAUUUCUUCCUCTT-3′
YTHDF1-siRNA#1 sense	5′- UGUACUUUAUUAUCUUGUCCUTT-3′
YTHDF1-siRNA#2 sense	5′- AAGUCAUUGUCAUGAACUGUATT-3′
NC-siRNA sense	5′-TTCTCCGAACGTGTCACGTTT-3′

Table S3. Association between SLC2A1-DT expression clinicopathological parameters of patients with LIHC

		SLC2A1-DT expression		
Parameter	No.	Low (n=179)	High (n=179)	*P*-value
**Age (years)**				
<=60	172	89	83	
>60	186	90	96	0.597
**Gender**				
Female	115	59	56	
Male	243	120	123	0.840
**T stage**				
T1 or T2	267	141	126	
T3 or T4	91	38	53	0.089
**N stage**				
N0	354	177	177	
N1	4	2	2	1
**M stage**				
M0	355	179	176	
M1	4	0	3	0.246
**G grade**				
G1 or G2	229	124	105	
G3 or G4	129	55	74	0.048
**TNM stage**				
I + II	230	133	117	
III + IV	108	46	62	0.084

Table S4. Univariate and multivariate analyses of SLC2A1-DT level and overall survival (TCGA).

Variable	Univariate analysis			Multivariate analysis		
	HR	95% Cl	P	HR	95% Cl	P
Overall survival (n=358)						
Age (years)	1.234	0.865-1.760	0.245			
≤60 (n=172)						
>60 (n=186)						
Gender	0.820	0.571-1.180	0.285			
Female (n=115)						
Male (n=243)						
Stage	2.544	1.788-3.618	<0.001	1.616	0.738-3.540	0.230
Stage I or II (n=250)						
Stage III or IV (n=108)						
T stage	2.623	1.836-3.745	<0.001	1.618	0.730-3.585	0.236
T1 or T2 (n=267)						
T3 or T4 (n=91)						
N stage	1.856	0.457-7.530	0.387			
N0 or NX (n=354)						
N1 (n=4)						

M stage	3.667	1.162-11.571	0.027	1.964	0.610-6.323	0.258
M0 or MX (n=355)						
M1 (n=3)						
G grade	1.105	0.769-1.588	0.591			
G1 or G2 (n=229)						
G3 or G4 (n=129)						
SLC2A1-DT (n=358)	3.535	1.670-7.481	<0.001	2.906	1.415-5.970	0.004

HR, hazard rate; CI, confidence interval.

Table S5 Mass spectrometry analysis of proteins pulled down by SLC2A1-DT

**SLC2A1-DT**	**Antisense**	**Differential protein**
KRT2	ACTB	DKFZp686J1497
ALB	ALB	KRT6B
KRT14	ALB GIG20	ATP5F1A
ANXA2	ANXA2	ATP5F1B
ATP5F1B	DKFZp586J151	ATP5B
DKFZp586J151	EFHD2	HBA1
DKFZp686J1497	EIF4A1	YWHAZ
EFHD2	EIF5A	HIST1H1T
EIF4A1	ETFA	RPLP0
EIF5A	H3F3B	HEL-S-123m
ETFA	HBB	HIST1H4J
H3F3B	HIST1H3A	TUBA1C
KRT10	HIST2H2AB	
HBA1	HIST2H3A	
HBB	HRNR S100A18	
KRT1	HSP90AA1	
HEL-S-123m	KRT1	
HIST1H1T	KRT1 KRTA	
HIST1H3A	KRT10	

HIST1H4J	KRT14
HIST2H2AB	KRT2
HIST2H3A	KRT5
HSP90AA1	KRT9
KRT5	RPSA
KRT6B	
KRT9	
RPSA	
TUBA1C	
YWHAZ	
RPLP0	
KRT1	
HRNR	
ATP5F1A	
ATP5B	
ALB GIG20	
ACTB	
